# Psoralen Derivatives as Inhibitors of *Mycobacterium tuberculosis* Proteasome

**DOI:** 10.3390/molecules25061305

**Published:** 2020-03-12

**Authors:** Kaja Rožman, Evan M. Alexander, Eva Ogorevc, Krištof Bozovičar, Izidor Sosič, Courtney C. Aldrich, Stanislav Gobec

**Affiliations:** 1 University of Ljubljana, Faculty of Pharmacy, Aškerčeva 7, SI-1000 Ljubljana, Slovenia; krozman@umn.edu (K.R.); eva.ogorevc@ffa.uni-lj.si (E.O.); kristof.bozovicar@ffa.uni-lj.si (K.B.); izidor.sosic@ffa.uni-lj.si (I.S.); 2 Department of Medicinal Chemistry, University of Minnesota, 308 Harvard Street Southeast, Minneapolis, MN 55455, USA; alexa589@umn.edu (E.M.A.); aldri015@umn.edu (C.C.A.)

**Keywords:** protein degradation, proteasome, *Mycobacterium tuberculosis*, psoralens, nonpeptidic proteasome inhibitors

## Abstract

Protein degradation is a fundamental process in all living organisms. An important part of this system is a multisubunit, barrel-shaped protease complex called the proteasome. This enzyme is directly responsible for the proteolysis of ubiquitin- or pup-tagged proteins to smaller peptides. In this study, we present a series of 92 psoralen derivatives, of which 15 displayed inhibitory potency against the *Mycobacterium tuberculosis* proteasome in low micromolar concentrations. The best inhibitors, i.e., **8**, **11**, **13** and **15**, exhibited a mixed type of inhibition and overall good inhibitory potency in biochemical assays. *N*-(cyanomethyl)acetamide **8** (*K_i_* = 5.6 µM) and carboxaldehyde-based derivative **15** (*K*_i_ = 14.9 µM) were shown to be reversible inhibitors of the enzyme. On the other hand, pyrrolidine-2,5-dione esters **11** and **13** irreversibly inhibited the enzyme with *K*_i_ values of 4.2 µM and 1.1 µM, respectively. In addition, we showed that an established immunoproteasome inhibitor, **PR-957**, is a noncompetitive irreversible inhibitor of the mycobacterial proteasome (*K_i_* = 5.2 ± 1.9 µM, *k*_inact_/*K_i_* = 96 ± 41 M^−1^·s^−1^). These compounds represent interesting hit compounds for further optimization in the development of new drugs for the treatment of tuberculosis.

## 1. Introduction

Protein turnover, a balance between the synthesis and degradation of proteins, is a fundamental biological process in all living organisms from prokaryotes to eukaryotes [1,2]. Cellular proteins are continuously synthesized and degraded at steady-state levels, thus enabling cells to dynamically adjust to internal and external stimuli, such as developmental reprogramming, environmental changes or the onset of disease states [1,3]. The rates of protein turnover have been optimized through evolution in line with biological function, and can vary from minutes to years in order to balance between energy-saving stability and dynamic flexibility [4,5].

**Figure 1:**
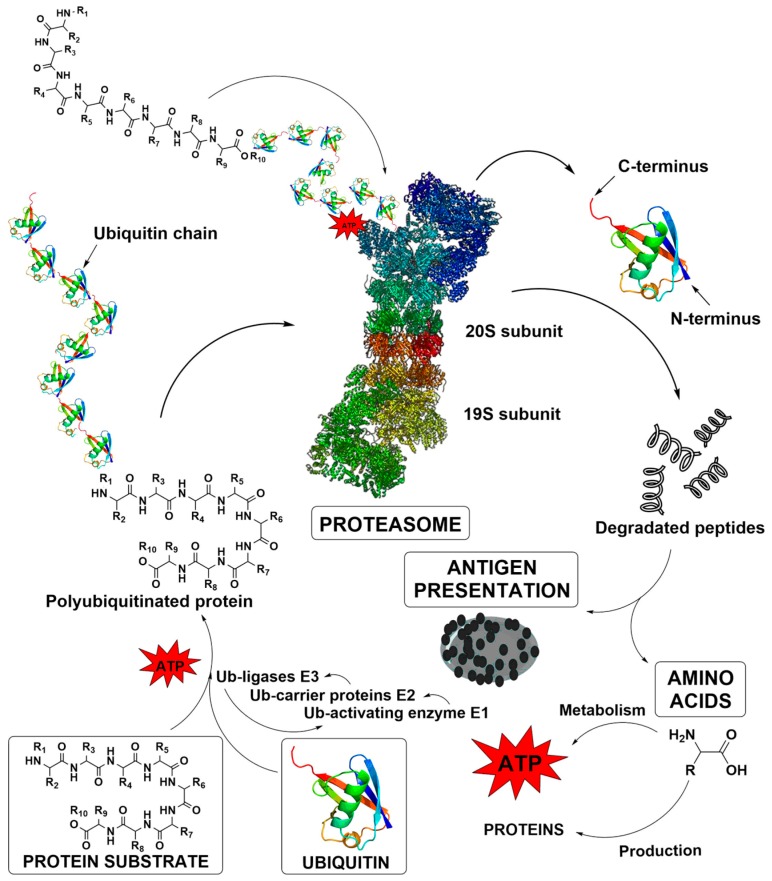
The ubiquitin – proteasome pathway in protein degradation [7,8,9]. The ubiquitination cascade is triggered by the ATP-mediated conjugation of a target protein with a single ubiquitin molecule by the Ub-activating enzyme E1. This tagged protein is then transferred to the Ub-carrier proteins E2 that subsequently forms a complex with Ub-ligases E3. The ligases recognize the protein and perform a sequence of ubiquitin additions until the final polyubiquitinated protein is formed. Finally, in human a rapid degradation of ubiquitinated protein by the proteasome is executed at six proteolytic sites of the enzyme. The degradation process results in 3 to 25 amino acids-long peptides, which are quickly digested into amino acids or can serve as antigen presentation molecules.

Proteasomes are multisubunit, barrel-shaped protease complexes that perform both ATP-dependent and -independent proteolysis, and thereby, play a pivotal role in protein turnover [6]. In eukaryotes, proteins are directed to the proteasome mostly through ubiquitination, a specific post-translational modification, in which a small protein called ubiquitin tags proteins for proteasomal degradation with the help of ubiquitin ligases in an ATP-dependent manner (Figure 1) [6]. In other words, protein degradation is a multistep procedure, which typically results in 3 to 25 amino acids-long peptides. These peptides have an extremely short existence as they are quickly digested into amino acids by the cytosolic endopeptidases and aminopeptidases. Accordingly, these amino acids can be later reutilized for the synthesis of new proteins or even metabolized to yield energy [7,8,9].

**Figure 2:**
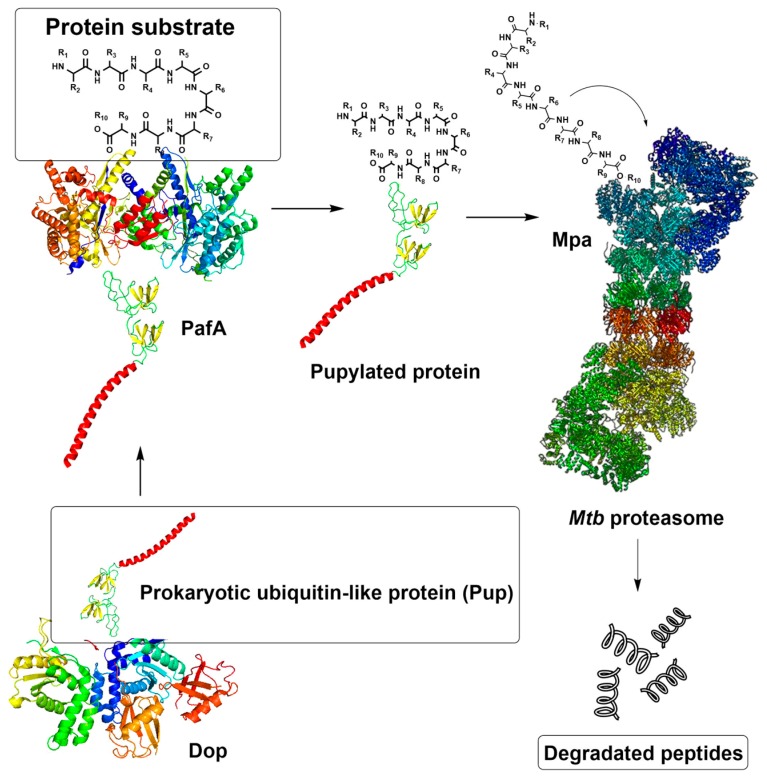
The pupylation pathway of protein degradation in *Mtb*.

In bacteria, proteasomes are an exception, as they are found only in the orders Nitrospirales and Actinomycetales; the latter includes the human pathogen *Mycobacterium tuberculosis* (*Mtb*), a causative agent of tuberculosis [10]. In these bacteria, proteins are directed to proteasomal degradation by pupylation, a mechanism analogous to ubiquitination in eukaryotes (Figure 2). Initially, the deaminase of Pup (Dop) converts the C-terminal glutamine of Pup (PupQ) to a glutamate (PupE), making it suitable for ligation [11]. Prokaryotic ubiquitin-like protein (Pup) is a functional analog of ubiquitin that is attached to specific lysine residues of substrate proteins by forming isopeptide bonds in a process catalyzed by the proteasome accessory factor A (PafA) [11,12]. The pupylated protein is then recognized by the *Mtb* proteasomal ATP-ase (Mpa) through a binding-induced folding mechanism that forms a unique α-helix. Afterward, Mpa delivers the Pup-substrate to the proteasome by coupling of ATP hydrolysis for proteasomal degradation [13]. In addition, there is an ATP-independent proteasome degradation route in *Mtb* that neither requires ATP nor interaction with Pup, and is mediated by proteasome accessory factor E (PafE, also referred to as Bpa) [14,15].

**Figure 3:**
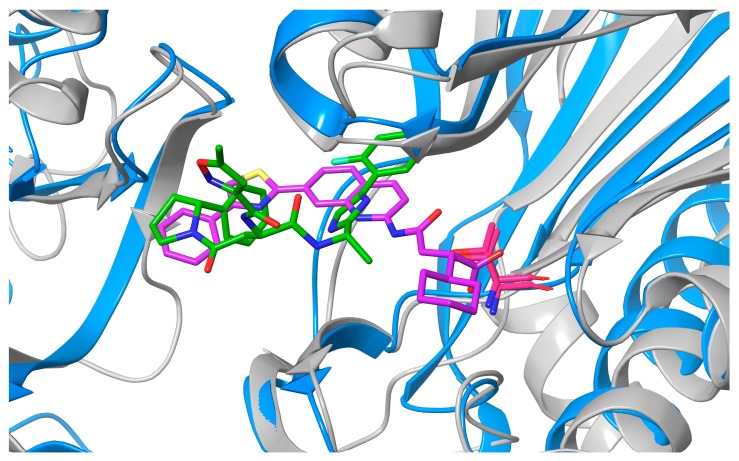
Superimposition of the β5i subunit of human IP (blue, PDB code 5M2B) and *Mtb* proteasome β subunit (grey, PDB code 6ODE) showing high similarity between the two enzymes and their active sites. The respective ligands Ro19 (purple) and B6 (green) are also presented. Catalytic threonine residues (Thr1) are red.

The overall architecture of the proteasome core is remarkably similar in all domains of life and consists of four stacked rings [16]. The two identical outer α-rings formed by seven α-subunits provide the entry point for the substrate, while the two identical inner β-rings, formed by seven β-subunits, hold the proteolytic activity [6]. In eukaryotes, there are seven types of α-subunits and seven types of β-subunits, with only three of the β-subunits (β1, β2 and β5) displaying proteolytic activities that are caspase-like, trypsin-like and chymotrypsin-like, respectively [17]. In contrast, the *Mtb* proteasome contains a single type of α- and β-subunit with broad substrate specificity, combining all these activities [18]. Binding of 7-amino-4-methylcoumarin-*N*-acetyl tripeptide substrates (AMC-P1-P2-P3) is directed in S1-3 binding pockets. Due to some features of S1 and S3 binding pockets, the proteasome favors substrates with a bulky tryptophan residue at the P1 position and either glycine or proline at the P3 position [10,19,20,21]. Importantly, the immunoproteasome (IP)—an isoform of eukaryotic proteasome, which is highly expressed in human hematopoietic cells, but also inducible in other type of cells in response to proinflammatory cytokines [22]—shares some significant characteristics with the *Mtb* proteasome. In both the human IP and *Mtb* proteasome, the S1 binding pocket is spacious and larger than that of constitutive human proteasome. Moreover, both the IP and *Mtb* proteasome prefer certain P1 amino acids in AMC-P1-P2-P3 substrates and small hydrophobic amino acids in P3 [19,20,23]. The structural similarity between chymotrypsin-like (β5i) subunit of the human IP and Mtb proteasome β subunit is presented in Figure 3.

Even though proteasomes in *Mtb* are not considered absolutely essential as they are in eukaryotes [24,25], their inactivation has been associated with some detrimental consequences for virulence, such as impaired survival in the mammalian host [26] and sensitivity to nitrosative stress [27]. As the hostile environment of *Mtb* is rapidly changing, it demands that the pathogen be highly metabolically flexible; an extensive protein turnover is a crucial process in responding to this challenge [28]. These findings bring *Mtb* proteasome among the prioritized targets for the treatment of tuberculosis, which is still one of the top ten causes of death worldwide and the leading cause of human mortality from an infectious disease [29]. Multidrug-resistant tuberculosis strains that do not respond to isoniazid and rifampicin, the two most powerful first-line anti-tuberculosis drugs, remain a global health safety threat. These strains can also be treated with second-line drugs, which are expensive, toxic and require long lasting chemotherapy. Moreover, extensively drug-resistant strains that do not respond to some of the most effective first- and second-line drugs are a growing problem, and often leave patients with few treatment options [29]. In 2018, an estimated 10 million new cases of tuberculosis occurred and approximately 1.5 million people died from this disease [29]. There is, therefore, a critical need for innovative antitubercular agents against new targets.

While the *Mtb* proteasome is an attractive target for the treatment of tuberculosis [20,30,31], the existence of human proteasomes poses a challenge for the development of selective inhibitors. The first identified inhibitors of the *Mtb* proteasome were indeed primarily developed to target the human proteasome, including bortezomib (Table 1) and epoxomicin, later upgraded to carfilzomib [27,32]. Bortezomib, carfilzomib and orally administered ixazomib are FDA-approved covalent peptidic inhibitors, used as therapeutics for multiple myeloma and mantle cell lymphoma that target both the constitutive proteasome and the IP [32], thus often resulting in severe toxicities [33]. As selective inhibition of IP is expected to attenuate the adverse effects, considerable efforts have been devoted to developing IP-specific inhibitors, resulting in some fairly advanced IP-selective compounds, such as **ONX-0914** (formerly **PR-957**, Table 1) and **KZR-616**, which are epoxyketone-based tripeptides [34]. Inhibitors with a peptidic backbone are prone to poor metabolic stability, and thus, exhibit low bioavailability [35,36]. Therefore, the introduction of novel, nonpeptidic IP inhibitors, such as quinolone-based compounds [37,38], oxathiazolones [39], piperlongumine analogues [40] and psoralens [41,42] has been of great importance. 

**Figure 4:**
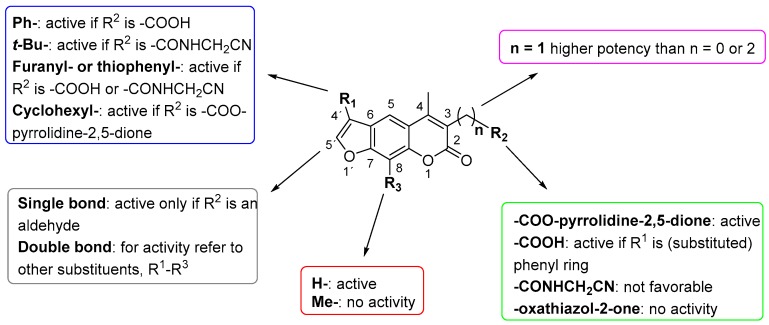
The summary of the SAR study of psoralen derivatives targeting the *Mtb* proteasome.

Nonpeptidic psoralen derivatives were identified using structure-guided virtual screening [41]. The initial virtual screening hit was first transformed into several nonpeptidic psoralen-based inhibitors, which were further optimized to irreversible inhibitors that specifically target the β5i subunit of IP. The psoralens (Figure 4) were shown to bind into the active site of IP subunit β5i either covalently or noncovalently. The docking studies demonstrated that the carboxylate group of the most potent reversible inhibitor (compound **16**, IC_50_ = 1.6 µM, Table S1) was positioned in the proximity of Thr1; therefore, various electrophilic groups were introduced into that portion of the molecule. The most potent compounds showed preferential inhibition of β5i over the β2i and β1i subunits of human IP, as well as over all three subunits of human constitutive proteasome [41]. Considering minimal off-target and cytotoxic effects of the series, along with the aforementioned substantial structural and mechanistic similarities between human IP and *Mtb* proteasome, we decided to further pursue the therapeutic potential of nonpeptidic psoralen derivatives by evaluating their inhibition of the *Mtb* proteasome.

## 2. Results and Discussion

Following the characteristics of some psoralen derivatives that selectively target the β5i subunit of human IP, we decided to explore the potential of this chemical class to inhibit the *Mtb* proteasome. Due to structural similarities between the IP and the *Mtb* proteasome, activity against the mycobacterial proteasome from psoralens would be expected. In this SAR study (Figure 4), we demonstrated that many psoralens indeed inhibit the *Mtb* proteasome. The inhibitory data for the most potent psoralens against the *Mtb* proteasome is presented in Table 1, and the complete results of the enzymatic assays are presented in the Tables S1 and S2.

**Figure 5:**
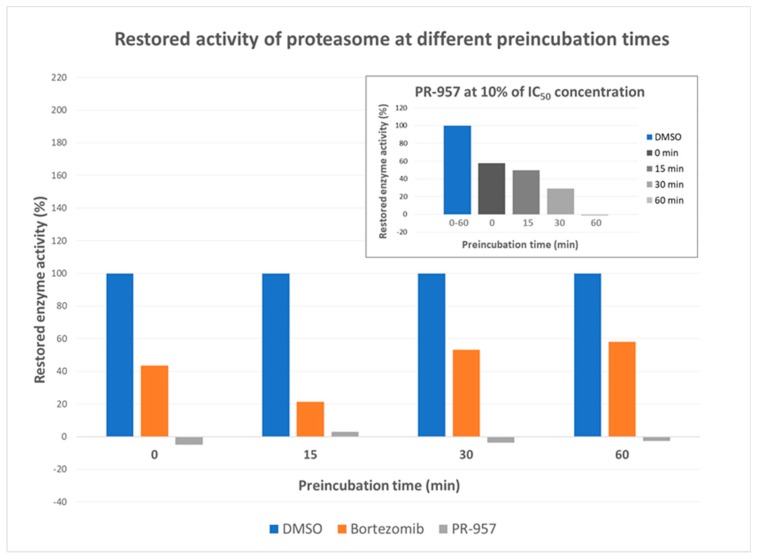
Dilution assay: Enzyme recovery after preincubation with the inhibitor for 0, 25, 30 or 60 min was observed and compared to DMSO control. Over longer preincubation time bortezomib caused significant loss of enzyme activity (up to approximately 45%) compared to the DMSO control at concentrations equal or higher than the inhibitor’s IC_50_ value. The recovery of proteasome seems to be slowly increasing with longer preincubation time. On the other hand, PR-957 completely inhibited the enzyme at higher concentrations regardless of the preincubation time. However, a time-dependent inhibition was observed at lower concentrations (top right graph).

Bortezomib was used as a positive control in the assays and, as expected, this boronate inhibited Mtb proteasome with high potency having IC_50_ and K_i_ values of 110 ± 30 nM and 133 ± 5 nM, respectively (Figures S2–S4). Furthermore, we evaluated compound **PR-957**, widely used as a selective β5i inhibitor of the human IP, for the inhibition of the *Mtb* proteasome; it proved to be less potent with IC_50_ and K_i_ values of 2.2 ± 1.0 µM and 5.2 ± 1.9 µM, respectively (Figures S5–S7). Interestingly, **PR-957** exhibited noncompetitive inhibition (k_inact_/K_i_ = 96 ± 41 M*^−^*^1^**·**s*^−^*^1^), unlike bortezomib, that showed mixed type of inhibition. Further, bortezomib inhibited the *Mtb* proteasome in a “slow/partially” reversible manner (Table 1 and Figures S21 and S22), while **PR-957** irreversibly inhibited the *Mtb* proteasome (Table 1 and Figures S23 and S24). To clarify, after 0-, 15-, 30- or 60-min preincubation of the enzyme with bortezomib (5 × IC_50_ concentration) at 25 °C, approximately 55% of the proteasome activity was retained after the catalytic reaction was initiated by the addition of the peptidic substrate Suc-LLVY-AMC (Figure 5). On the other hand, no significant recovery of proteasome activity was observed after treatment with **PR-957** at a concentration of the compound equal or higher than its IC_50_, but showed the time-dependent inhibition (from 45% to a 100% at increasing time of preincubation) at 10% of the IC_50_ concentration (Figure 5 and Figure S23). It is important to note that the activity of the *Mtb* proteasome decreases significantly after an hour of preincubation of the enzyme with DMSO and without the inhibitor. Thus, 30 min preincubation of the enzyme with the inhibitor or DMSO was chosen as optimal. The complete data can be found in Supplementary data (Figures S21–S32).

We evaluated 92 psoralens against the *Mtb* proteasome to determine the SAR that governs inhibition and selectivity (Table 1 and Table S1). Among the psoralen library, 15 showed inhibitory potencies below 40 µM (2 µM < IC_50_ < 40 µM) against the *Mtb* proteasome. All assay where performed in the presence of 50 μM of the substrate Suc-LLVY-AMC, for which *K*_m_ value of 60 ± 15 µM was determined (literature value [18]: 56 ± 15 µM). The SAR data is succinctly summarized in Figure 4. Firstly, neither the methyl group at position 4 nor at position 8 of the psoralen scaffold are imperative for the activity (Figure 4). In fact, the methyl group at the position 8 was mostly associated with the loss of potency (Table S1). The length of alkyl chain at position 3 of the psoralen allows for some flexibility, but a one methylene linker is preferred. The substituent R_2_ is extremely tolerant to modification and analogs containing a carboxylic acid (compounds **1**–**6**), *N*-(cyanomethyl)acetamide (compounds **7**–**10**), ester with pyrrolidine-2,5-dione (compounds **11**–**14**) or carboxaldehyde (compound **15**) at R_2_ were potent, with the exception of the derivative containing a 1,3,4-oxathiazol-2-one group (compounds **88**–**90**, Supplementary data). In addition, it was found that the substituent R_1_ at position 4′ of the furan ring can vary. For example, R_1_ can be a (substituted) phenyl, furanyl, (substituted) thiophene ring, *tert*-butyl or cyclohexyl; however, the potencies of these compounds were influenced by the substituent R_2_. The interrelations between substituents in those two positions are described in Figure 4.

After the SAR study was concluded, we performed additional biochemical analyses for selected compounds to determine the modality of inhibition. We observed mixed-type inhibition for the four most potent inhibitors, i.e., **8**, **11**, **13** and **15** (Table 1 and Figures S8–S19). Further analysis of these four compounds showed reversible inhibition for the *N*-(cyanomethyl)acetamide **8** and the aldehyde **15** (Figures S25, S26, S31 and S32), but only partially reversible inhibition for the pyrrolidine-2,5-diones **11** and **13** (Figures S27–S30). Interestingly, these two compounds showed reversible inhibition at concentrations ≤ IC_50_, but at concentrations > IC_50_, the enzyme activity was only partially recovered (up to 35%), which could imply that compounds **11** and **13** interfere with the substrate binding, or that they cause protein aggregation at higher concentrations. Compound **15** is an aldehyde and a racemate that showed good inhibitory potency (IC_50_ = 5.8 ± 2.1 µM, *K*_i_ = 14.9 ± 45.0 µM); this could suggest that binding to half of the binding sites of the proteasome is sufficient to fully inhibit function (Hill coefficient equals 0.55). On the other hand, compounds **8**, **11** and **13** bind to the *Mtb* proteasome in a 1:1 ratio. However, we must not forget that there are 14 catalytic ß-subunits with 14 active sites that represent potential binding sites for the inhibitors. Therefore, other psoralens with higher Hill coefficients should not be immediately characterized as nonspecific. Reversible inhibitor **8** showed inhibitory potency in lower micromolar range, i.e., IC_50_ value of 3.8 ± 1.5 µM and *K*_i_ = 5.6 ± 20.8 µM, as did both partially reversible inhibitors **11** (IC_50_ = 8.8 ± 1.0 µM, *K*_i_ = 4.2 ± 2.1 µM) and **13** (IC_50_ = 3.2 ± 0.3 µM, *K*_i_ = 1.1 ± 0.9 µM). These compounds can, therefore, be selected as important hit inhibitors that possess a good on-target activity and should be considered for further optimization in the future.

Finally, since these compounds were previously described as inhibitors of human IP [41,42], a possible selectivity trend for either *Mtb* proteasome or human IP was evaluated. Both bortezomib and **PR-957** appear to be selective towards the human IP, i.e., approximately 30- and 300-fold, respectively. Further, derivatives with carboxylic acid at position 3 and with variations at positions 4′ and 8 (**1**–**6**, **16**–**39**, Table 1 and Table S1) in general showed higher potency against the human IP compared to the *Mtb* proteasome, except compounds **1**–**5**, which showed better activity against the *Mtb* enzyme. These compounds are all furnished with a *para*-substituted phenyl ring at 4′ (Figure 4). Such a substitution pattern seems to favor binding to the *Mtb* proteasome as opposed to the human IP. Although no major conclusions could be drawn regarding the reason for this, it was clear that the selectivity towards one or the other proteasome was not influenced by the length of the side methylene linker at position 3, or the methyl substituent at position 8. The nitrile-based compounds (**7**–**10**, **40**–**65**) were described as being less potent against the ß5i of human IP than compound **16** (*K*_i_ = 1.6 ± 0.7 µM against the ß5i of IP, Table S1), which was the first lead compound derived from virtual screening [41]. Interestingly, **16** did not inhibit the *Mtb* proteasome (Table S1). Similarly, most of the nitrile-based compounds also showed poor inhibition of the *Mtb* proteasome (Table S1), except compounds **7**–**10**, which showed selective inhibition of the *Mtb* enzyme in the micromolar range (Table 1). 

The majority of esters with pyrrolidine-2,5-dione (**11**–**14**, **66**–**79**) are nonselective, as they inhibit both the IP and the *Mtb* proteasome in a similar concentration range (Table 1 and Table S1), with few exceptions, i.e., compounds **66**–**69**, **72**, **73** and **79** (Table S1), which preferably inhibit human IP. Several other substitutions at position 3 (compounds **80**–**90**, Table S1) yielded inactive compounds against the *Mtb* proteasome; however, oxathiazolones **88**–**90** showed excellent activity against the human IP in a nanomolar range (IC_50_ value of 13–20 nM) [41]. Thus, these three compounds showed high selectivity towards the human enzyme. The only partially saturated psoralen derivative that showed inhibition of *Mtb* proteasome was an aldehyde **15**, which inhibited *Mtb* proteasome and showed reversible mixed type mode of action with *K*_i_ value of approximately 15 µM (Table 1).

To conclude, psoralens generally show preference for the human IP; however, several derivatives (compounds **1**–**5** and **7**–**10**) selectively inhibited the *Mtb* proteasome in a low micromolar range, and could, considering the fact that this chemotype has never been described to inhibit *Mtb* proteasome, therefore, be used as leads for further optimization towards improved antimycobacterial activity.

## 3. Materials and Methods 

### 3.1. Isolation of the Enzyme

The pACYCDuet-1 vector encoding *Prc*AB plasmid was transformed into BL21(DE3) competent cells (C2527H) [43] and the *Mtb* wild type proteasome was expressed and purified as described [18]. The plasmid *Prc*AB consists of pT7/logOp, the origin of plasmid replication p15A, chloramphenicol resistance gene, *lacI* gene and expresses PrcA and His-tagged PrcB. The image of the purified enzyme is shown in Figure S1 in *Supplementary data*.

### 3.2. Enzymatic Assays

Enzymatic assays with the *Mtb* proteasome were performed in 384-well Corning low volume, round bottom black plates by measuring the fluorescence (λ_ex_ = 360 nm, λ_em_ = 460 nm) on a plate reader Biotek Synergy H1 equipped with Gen5 software. The fluorescence forming reaction (hydrolysis of substrate Suc-LLVY-AMC) was continuously monitored under initial velocity conditions for 90 min at 37 °C.

Stock solutions of the compounds (1 or 10 mM) were prepared in DMSO. The final concentration of DMSO in the assay was 2% v/v. First, 0.2 μL of the compound solution was preincubated with 7.8 μL of the *Mtb* proteasome (7 nM) in 20 mM HEPES buffer, pH = 7.5 with 0.5 mM EDTA, for 30 min at 37 °C. The enzymatic reaction was initiated by the addition of 2 μL Suc-LLVY-AMC (50 μM) and the reaction was monitored for an additional 90 min at 37 °C. Control reactions were carried out at the same conditions as described above, but without the inhibitor and with 2% *v*/*v* DMSO. Bortezomib was used as a positive control. The inhibitory potency of each compound was expressed as the percentage of *Mtb* proteasome activity inhibition (initial velocity of the reaction) with respect to the control reaction without the inhibitor (residual activity, RA). All experiments were repeated twice at different times with standard deviations within ± 10%. The results are presented in Tables S1 and S2. IC_50_ values were determined by varying the concentration of inhibitor in the same conditions as described above. All experiments were done in triplicate. The results are presented in Table 1.

#### 3.2.1. Determination of K_i_ and K_m_ Values

For a positive control, i.e. bortezomib, and compounds **8**, **11**, **13** and **15** inhibition constants (*K*_i_) were measured under similar conditions to those described above, but with various concentrations of the substrate Suc-LLVY-AMC (12.5, 25, 50, 100, 250, 500 µM). The concentrations of **11**, **13** and **15** were 0, 0.56, 0.81, 1.15, 1.65, 2.35, 4.80, 9.80 and 20 µM. Further, the concentrations for compound **8** were 0, 1.65, 2.35, 4.80, 9.80 µM and for bortezomib 0, 0.28, 0.40, 0.58, 0.82, 1.18, 2.40, 4.90 and 10 µM. All experiments were performed in triplicate. The resulting data were analyzed using the GraphPad Prism 6 software [44] and were fitted to the software provided models for competitive, noncompetitive, uncompetitive and mixed type enzyme inhibition. The mode of inhibition and *K*_i_ values were chosen from the best ranking model, as determined by the software. Representative graphs (Michaelis-Menten, Dixon and Lineweaver-Burk plots) depicting the data fit for the best ranking model of inhibition for all compounds are shown in the *Supplementary data* (Figures S2–S19).

Additionally, the *K*_m_ of the substrate Suc-LLVY-AMC was calculated based on the initial velocity of the enzymatic reaction using 10 nM *Mtb* proteasome at various concentration of the substrate (12.5, 25, 50, 100, 250, 500 µM). The representative Lineweaver-Burk plots (1/[v] vs. 1/[S]) are presented in *Supplementary data* (Figure S20).

#### 3.2.2. Dilution Assays

The *Mtb* proteasome (1 µM) at 100-fold of final concentration (10 nM) was incubated with the inhibitor at various concentrations (0.1-, 1- and 2- or 5-fold concentration the IC_50_) for a certain amount of time (0, 15, 30 and 60 min) at room temperature (volume of the stock reaction mixture, 10 µL). This mixture (1 µL) was diluted 100-fold with the substrate (50 µM substrate in 20 mM HEPES, pH = 7.5 with 0.5 EDTA) to a final volume of 100 µL. Control reactions were carried out under the same conditions as those described above, but without the inhibitor and with 1% *v*/*v* DMSO. In the case of reversible inhibitors, the enzyme recovery is expected to be more than 90%. All experiments were performed in duplicates with standard deviations within ± 10%, except for DMSO control reactions, that were performed in triplicate with standard deviations within ± 10%, and were repeated twice.

#### 3.2.3. Determination of k_inact_/K_i_ values for PR-957

The kinetic assays were conducted as previously described in *Enzymatic assays*. Briefly, 7.8 μL of the *Mtb* proteasome (final concentration of 7 nM) in 20 mM HEPES buffer, pH = 7.5 with 0.5 mM EDTA was added to 0.2 μL of the compound solution at 100x final concentration in DMSO. The concentrations of **PR-957** used were 0, 1.15, 1.65, 2.35, 4.80, 9.80 and 20 µM. The enzymatic reaction was initiated by the addition of 2 μL Suc-LLVY-AMC (final concentration of 50 μM) and the reaction progress was determined on a BioTek Synergy HT microplate reader, by monitoring fluorescence at 460 nm (λ_ex_ = 360 nm) for 90 min at 37 °C. Values of *k_obs_* were derived from the fit of the data to the equation P = (v_i_/k_obs_)x [1 − exp(−*k*_obs_·t)] in GraphPadPrism. The data obtained were afterwards plotted against inhibitor concentration, to obtain *k*_inact_/*K*_i_ by fitting to the equation *k*_obs_ = *k*_inact_/(1 + *K*_i_/[I]). The representative plots are presented in *Supplementary data* (Figure S33–S36).

### 3.3. Compounds

Bortezomib was purchased from Sigma-Aldrich, and PR-957 was purchased from MedChemExpress. Compound purities were measured by the analytical reversed-phase HPLC on an Agilent 1100 LC modular system that was equipped with a photodiode array detector set to 254 nm. The experiments were performed on an Agilent Eclipse Plus C18 column (150 × 4.6 mm; 5 μm) at 25 °C, with a flow rate of 1.0 mL/min and a sample injection volume of 10 μL. An eluent system of A (0.1% TFA in H2O) and B (MeCN) was used according to the general method of: 0–16 min, 40% B → 90% B; 16–19 min, 90% B; 19–20 min, 90% B → 40% B) The purities of the test compounds used for the biological evaluations were >95%, as determined by HPLC, with the exception of compounds **1**, **59** and **83**, whose purities were 88.10%, 91.38% and 83.56%, respectively. 

## 4. Conclusions

Psoralen derivatives are known to inhibit the IP in human cells, and are therefore promising lead compounds in cancer therapy. In this study, we showed that these inhibitors are also active against the *Mtb* proteasome. We identified 15 psoralens from a library of 92 analogs that possessed IC_50_ values between 2 and 40 µM against the *Mtb* proteasome. The most potent inhibitors **8**, **11**, **13** and **15** showed potent inhibition in a fluorescence-based enzymatic assay, and they all exhibited a mixed type of inhibition. Compounds **8** and **15** were found to be reversible inhibitors with a mixed modality of inhibition with *K*_i_ values of 5.6 µM (α = 0.19) and 14.9 µM (α = 0.22), respectively. On the other hand, at concentrations higher than their IC_50_ values, only little *Mtb* proteasome activity was restored (<35%) when treated with the pyrrolidine-2,5-dione esters **11** and **13**, suggesting a partially reversible inhibition. However, they showed clear, reversible inhibition at lower concentrations with *K*_i_ values of 4.2 µM (α = 6.67) and 1.1 µM (α = 6.94 × 10^^16^), respectively. Therefore, these compounds show potential for further optimization in the development of new drugs for the treatment of tuberculosis. 

## Figures and Tables

**Table 1:** The inhibitory potencies of selected psoralen derivatives against the *Mtb* proteasome. Inhibition data for IP is added to evaluate selectivity profile of compounds.

Compound	Chemical Structure	IC_50_ (µM) and (Hill Coefficient)	*K*_i_ and Type of Inhibition ^a^	RA ^b^ at 10 µM (%) or *K*_i_ or IC_50_ (µM) against ß5_i_ of IP (refs [41,42])
**Bortezomib**	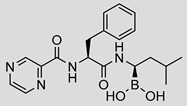	0.11 ± 0.03 (0.76)	*K*_i_ = 133 ± 5 nM Mixed inhibition (R^2^ = 0.94, α = 0.85) (Slow) reversible inhibition	IC_50_ = 0.004 µM (ref [35])
**PR-957**	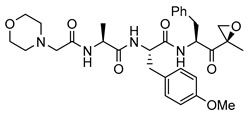	2.2 ± 1.0 (0.91)	*k*_inact_/*K*_i_ = 96 ± 41 M^−1^·s^−1^ *K*_i_ = 5.2 ± 1.9 µM Noncompetitive inhibition (R^2^ = 0.83) Irreversible inhibition	IC_50_ = 0.015 ± 0.002 µM (ref [41])
**1**	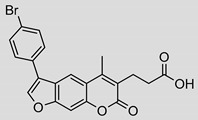	39 ± 7 (0.69)	ND	*K*_i_ = 137 ± 33 µM
**2**	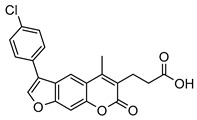	15 ± 2 (1.75)	ND	83%
**3**	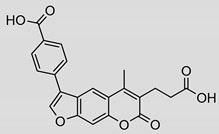	31 ± 5 (1.22)	ND	53%
**4**	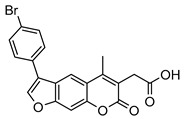	4 ± 3 (1.59)	ND	*K*_i_ = 12.7 ± 3.7 µM
**5**	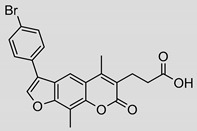	17 ± 3 (0.70)	ND	93%
**6**	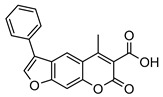	2.2 ± 0.3 (0.70)	ND	82%
**7**	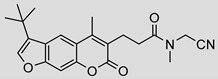	8 ± 5 (0.42)	ND	100%
**8**	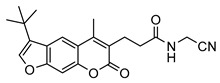	3.7 ± 1.5 (1.05)	*K*_i_ = 5.6 ± 20.8 µM Mixed inhibition (R^2^ = 0.54, α = 0.19) Reversible inhibition	100%
**9**	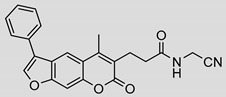	2 ± 2 (2.27)	ND	66%
**10**	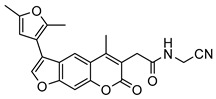	3 ± 2 (4.46)	ND	100%
**11**	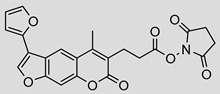	8.8 ± 1.0 (0.90)	*K*_i_ = 4.2 ± 2.1 µM Mixed inhibition (R^2^ = 0.91, α = 6.67) (Partially) reversible inhibition	IC_50_ = 0.94 ± 1.1 µM
**12**	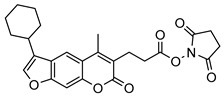	5 ± 2 (1.05)	ND	IC_50_ = 1.8 ± 0.4 µM
**13**	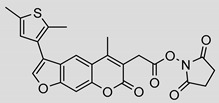	3.2 ± 0.3 (0.90)	*K*_i_ = 1.1 ± 0.9 µM Mixed inhibition (R^2^ = 0.52, α = 6.94 × 10^^16^) (Partially) reversible inhibition	IC_50_ = 4.4 ± 0.1 µM
**14**	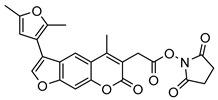	9 ± 5 (2.42)	ND	IC_50_ = 6.9 ± 2.1 µM
**15**	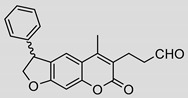	5.8 ± 2.1 (0.55)	*K*_i_ = 14.9 ± 45.0 µM Mixed inhibition (R^2^ = 0.77, α = 0.22) Reversible inhibition	ND

^a^*K*_i_ determined with GraphPad Prism by fitting the data to several inhibition models (mixed, competitive, noncompetitive, uncompetitive). The best scoring model was further examined by creating Dixon, Lineweaver-Burk and Michaelis-Menten plots. *K*_m_ for Suc-LLVY-AMC is 60 ± 15 µM (see *Supplementary data* for details). ND, not determined. ^b^ The data were calculated as residual activities (RAs) of β5i in the presence of 10 μM of each compound (standard errors for RAs were < 15%).

## Supplementary Materials

The following are available online at https://www.mdpi.com/1420-3049/25/6/1305/s1. Figure S1: Gel image of purified Mtb proteasome, Figures S2–S32: Enzyme kinetics plots, Table S1: Chemical structures, molecular weights and residual activities of inactive psoralens.
