# Supplementary material for: Psoralen Derivatives as Inhibitors of Mycobacterium tuberculosis Proteasome
Source: Molecules. 2020 Mar 12;25(6):1305. doi: 10.3390/molecules25061305 (PMC7144120; doi:10.3390/molecules25061305)
Supplement: Supplementary file 1 [file molecules-25-01305-s001.pdf]

molecules

Structure-activity relationships study of psoralen derivatives as inhibitors of *Mycobacterium tuberculosis* proteasome

Kaja Rožman [1,2], Evan M. Alexander [2], Eva Ogorevc [1], Krištof Bozovičar [1], Izidor Sosič [1], Courtney C. Aldrich [2] and Stanislav Gobec [1,*]

Supplementary data

Contents

1. Protein expression and isolation 2
2. Enzymatic assay 2
3. Determination of K_i and K_m values 11
   a. Bortezomib 11
   b. **PR-957** 12
   c. Compound **8** 14
   d. Compound **11** 16
   e. Compound **13** 17
   f. Compound **15** 19
   g. Determination of K_m value for Suc-LLVY-AMC 20
4. Dilution assay 21
   a. Bortezomib 21
   b. **PR-957** 22
   c. Compound **8** 23
   d. Compound **11** 24
   e. Compound **13** 25
   f. Compound **15** 26
5. Analytic and synthetic data for compounds .... **Error! Bookmark not defined.**

1. Protein expression and isolation

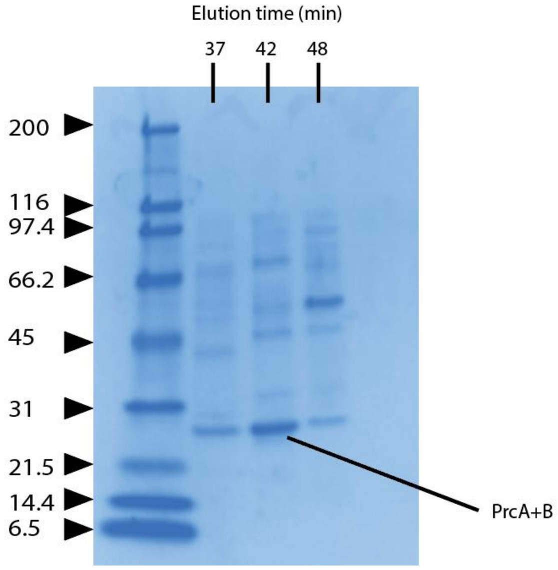

Figure S1. Gel image of purified Mtb proteasome with a clear band at 26-27 kDa representing proccessed *α* and ß subunits.

2. Enzymatic assay

Table S1. Chemical structures, molecular weights and residual activities of inactive psoralens.

Compound	Chemical structure	M (g/mol)	RA [a] at 100 μM against *Mtb* (%)	RA [a] at 10 μM (%) or K_i or IC_{50} (μM) against ß5$_i$ of IP [b]
**16**	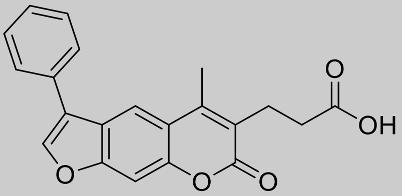	348.35	72%	K_i = 1.6 ± 0.7 μM
**17**	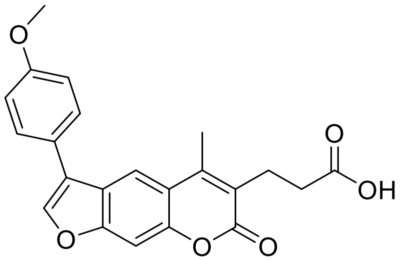	378.37	100%	84%
**18**	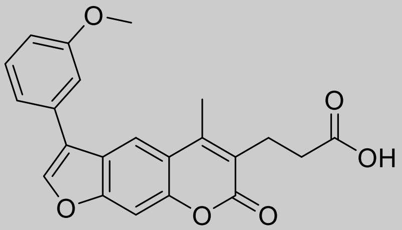	378.37	100%	K_i = 29.6 ± 3.9 μM

**19**	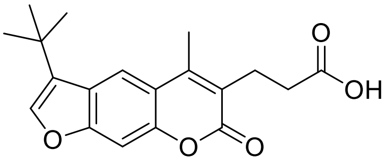	328.36	95%	83%
**20**	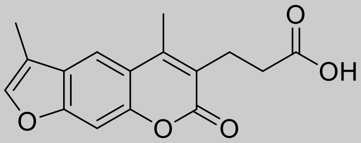	286.28	93%	94%
**21**	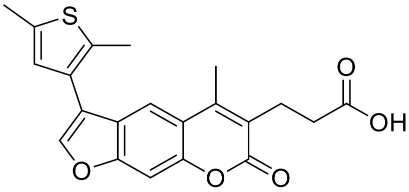	382.43	99%	K_i = 52.3 ± 18 μM
**22**	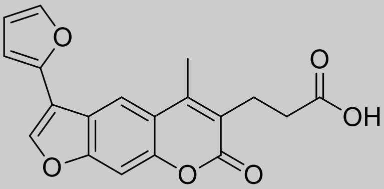	338.31	55%	K_i = 116 ± 27 μM
**23**	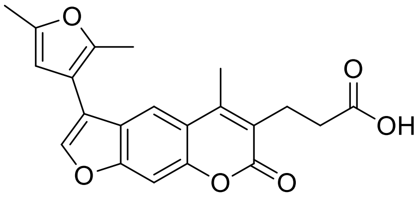	366.36	76%	100%
**24**	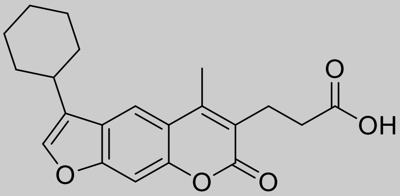	354.40	71%	102%
**25**	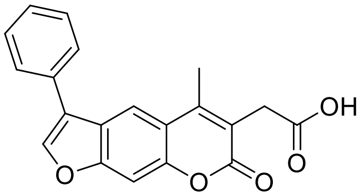	334.32	96%	K_i = 2.4 ± 0.4 μM
**26**	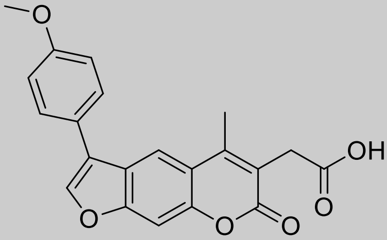	364.35	50%	K_i = 8.2 ± 0.5 μM
**27**	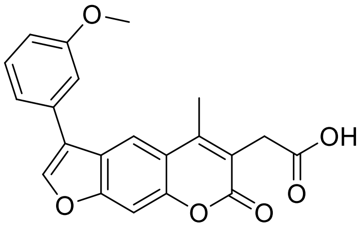	364.35	98%	K_i = 7.0 ± 0.2

**28**	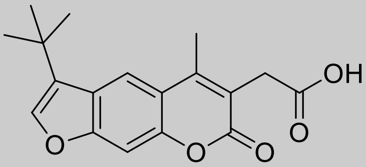	314.34	97%	85%
**29**	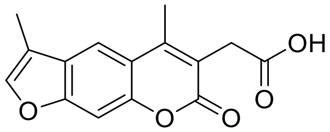	272.26	61%	88%
**30**	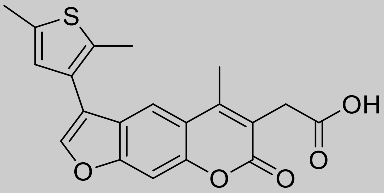	368.40	93%	K_i = 36.5 ± 7.1 μM
**31**	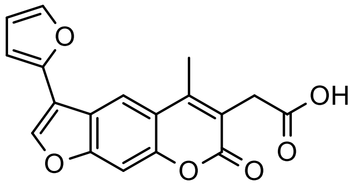	324.28	79%	K_i = 89.3 ± 2.0 μM
**32**	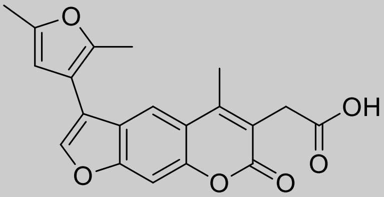	352.34	100%	K_i = 351 ± 48 μM
**33**	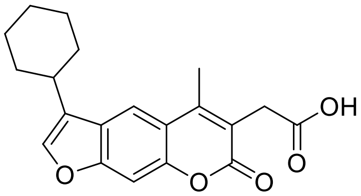	340.37	90%	K_i = 198 ± 21 μM
**34**	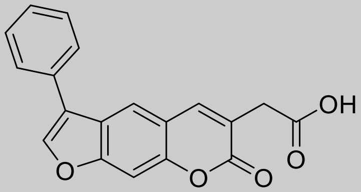	320.30	54%	K_i = 3.8 ± 1.1 μM
**35**	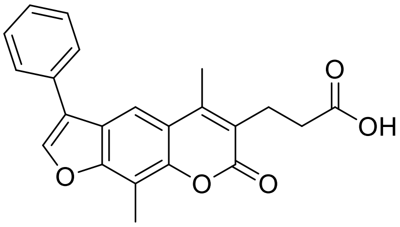	362.38	100%	K_i = 43.1 ± 15 μM
**36**	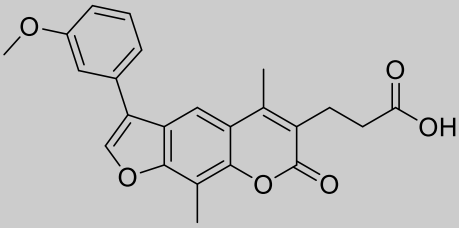	392.40	87%	72%

**37**	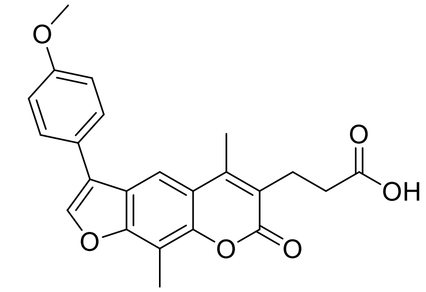	392.40	87%	87%
**38**	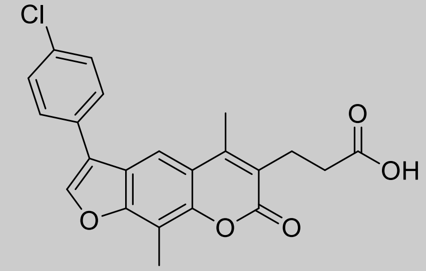	396.82	47%	89%
**39**	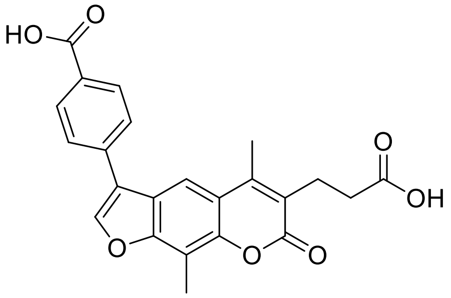	406.38	98%	ND
**40**	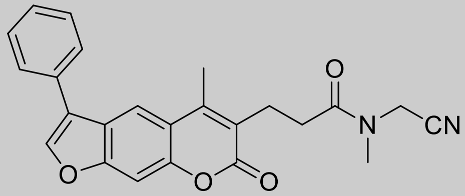	400.43	100%	92%
**41**	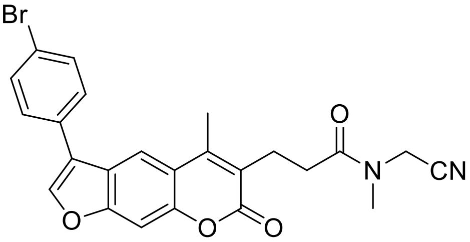	479.32	91%	107%
**42**	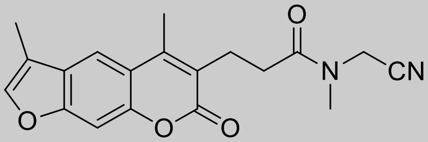	338.36	99%	93%
**43**	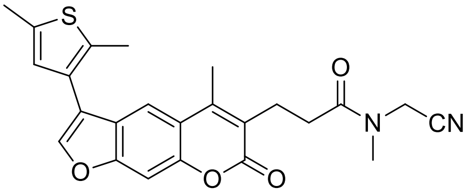	434.51	55%	107%
**44**	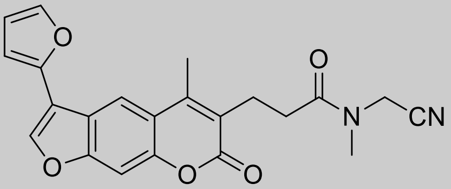	390.39	82%	86%

**45**	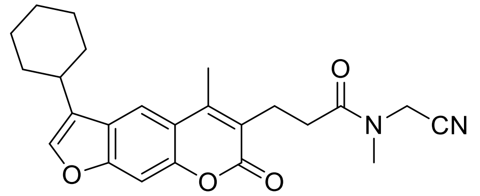	406.47	92%	111%
**46**	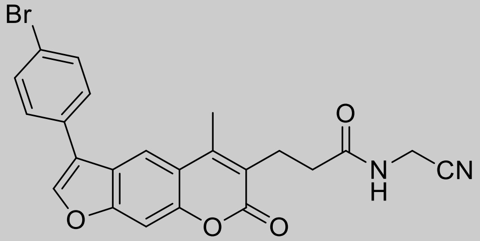	465.30	82%	107%
**47**	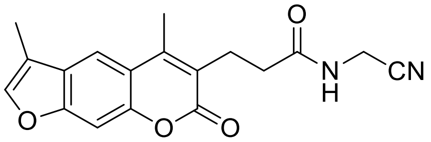	324.33	95%	105%
**48**	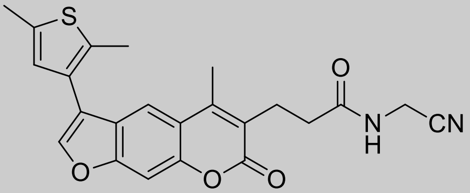	420.48	100%	109%
**49**	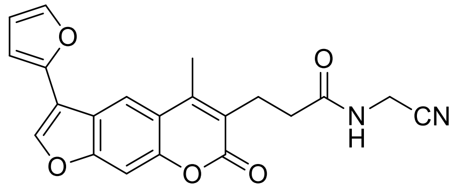	376.36	100%	97%
**50**	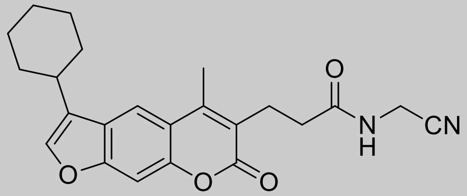	392.45	96%	102%
**51**	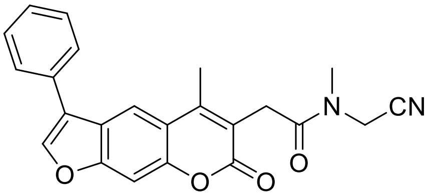	386.40	93%	97%
**52**	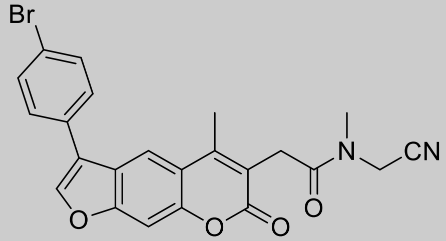	465.30	70%	86%
**53**	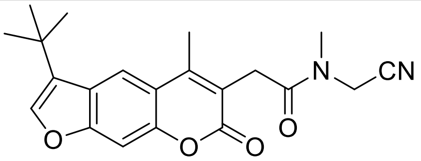	366.41	97%	96%
**54**	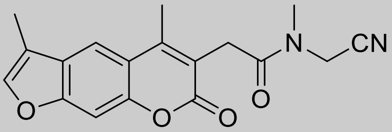	324.34	95%	99%

**55**	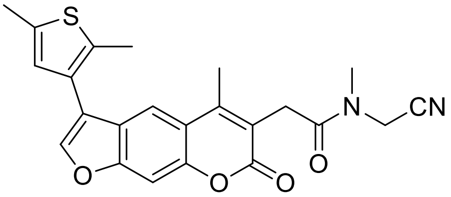	420.48	88%	103%
**56**	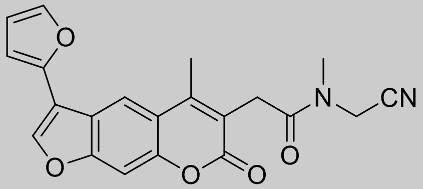	376.36	53%	103%
**57**	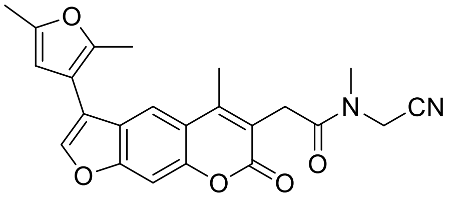	404.42	100%	96%
**58**	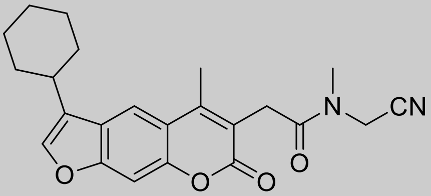	392.45	100%	101%
**59**	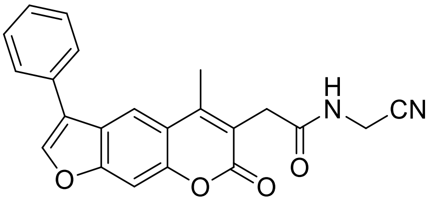	372.37	92%	101%
**60**	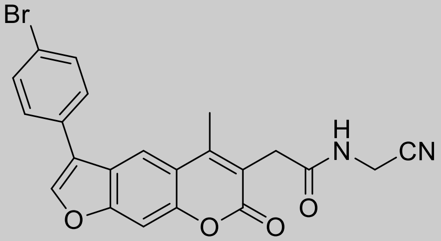	451.27	87%	105%
**61**	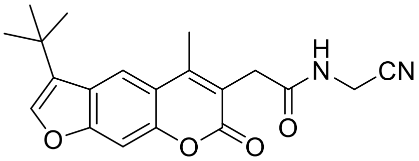	352.38	92%	91%
**62**	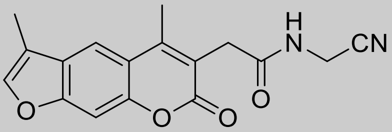	310.31	60%	ND
**63**	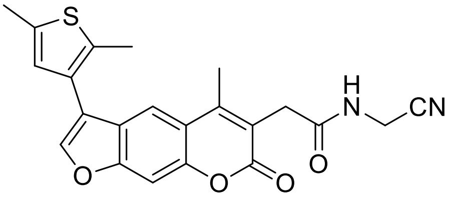	406.45	95%	101%

**64**	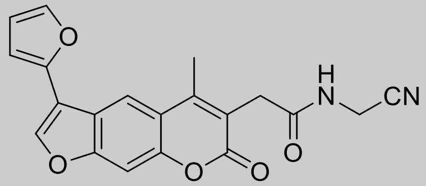	362.34	91%	98%
**65**	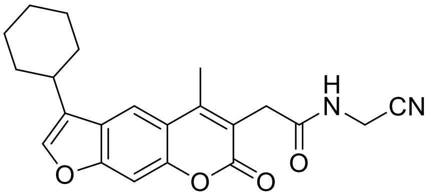	378.42	91%	99%
**66**	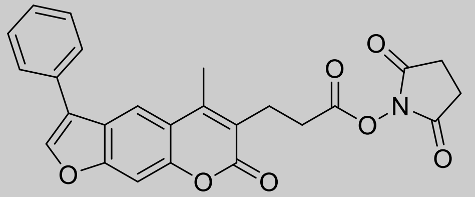	445.42	66%	IC_{50} = 0.009 ± 0.002 μM
**67**	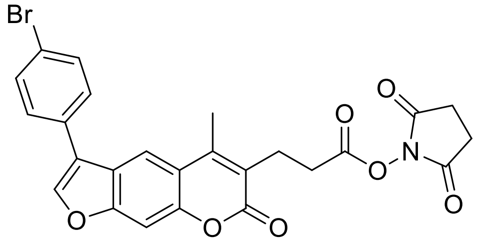	524.32	96%	IC_{50} = 1.9 ± 0.9 μM
**68**	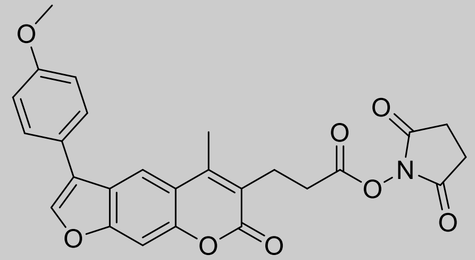	475.45	93%	IC_{50} = 0.64 ± 0.13 μM
**69**	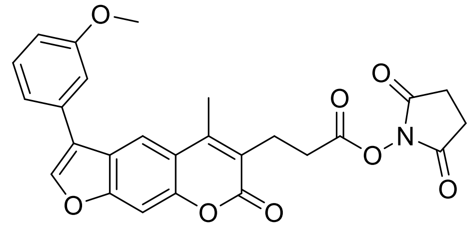	475.45	72%	IC_{50} = 0.93 ± 0.57 μM
**70**	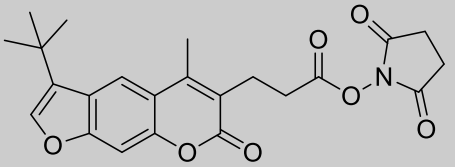	425.43	70%	41%
**71**	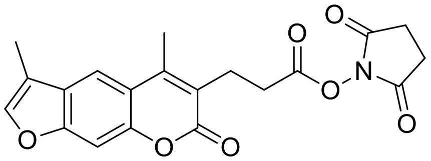	383.35	97%	66%
**72**	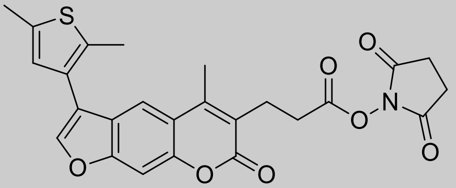	479.50	95%	IC_{50} = 1.8 ± 0.8 μM

**73**	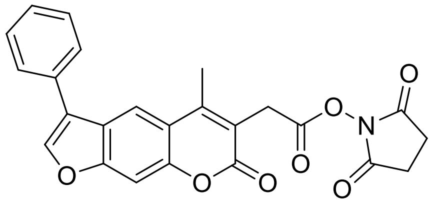	431.29	79%	IC_{50} = 6.36 ± 0.61 μM
**74**	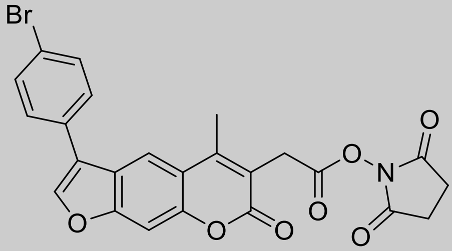	510.29	61%	101%
**75**	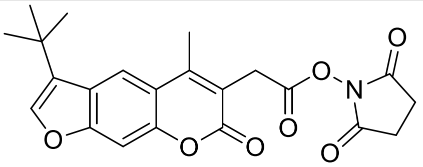	411.40	88%	53%
**76**	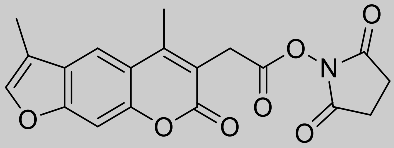	369.33	53%	101%
**77**	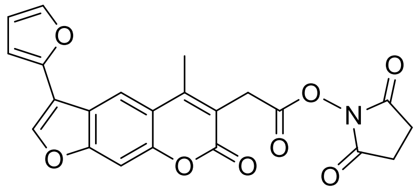	421.36	56%	44%
**78**	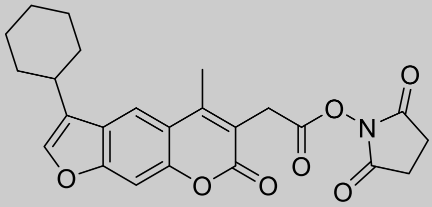	437.44	98%	62%
**79**	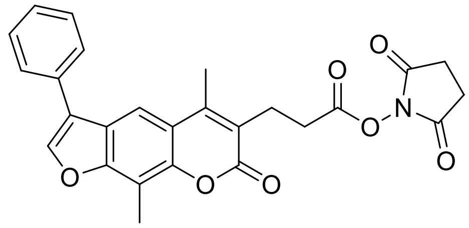	459.45	100%	IC_{50} = 3.26 ± 1.49 μM
**80**	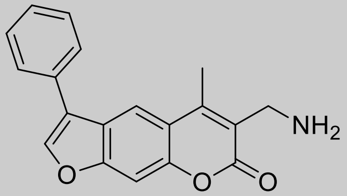	305.33	55%	ND
**81**	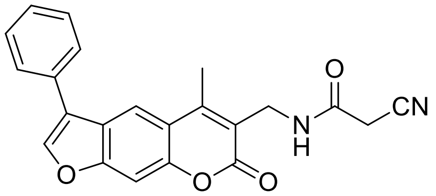	372.38	100%	101%

**82**	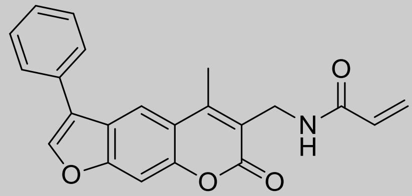	359.37	68%	90%
**83**	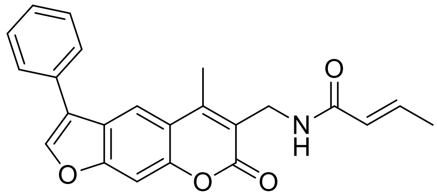	373.40	52%	116%
**84**	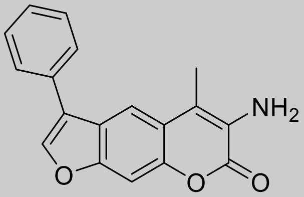	291.31	100%	88%
**85**	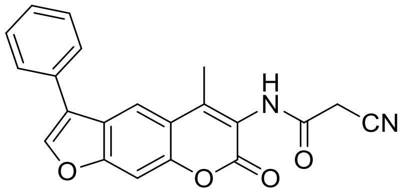	358.35	93%	115%
**86**	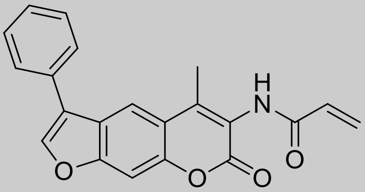	345.35	95%	IC_{50} = 20.51 ± 1.36 μM
**87**	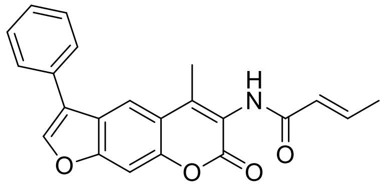	359.37	93%	102%
**88**	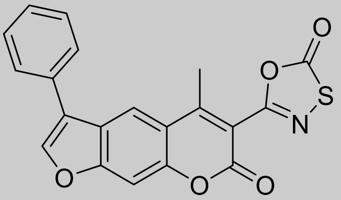	377.37	84%	IC_{50} = 0.013 ± 0.004 μM
**89**	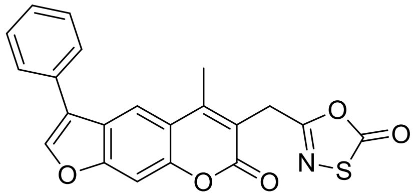	391.40	65%	IC_{50} = 0.12 ± 0.05 μM
**90**	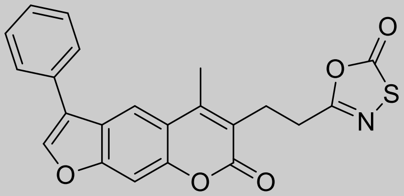	405.42	69%	IC_{50} = 0.20 ± 0.04 μM

**91**	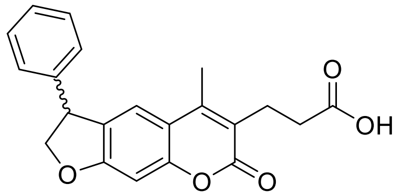	350.36	95%	94%
**92**	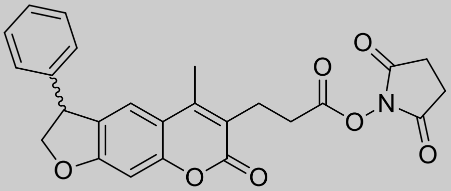	447.44	95%	36%

[a] The data were calculated as residual activities (RAs) in the presence of 10 μM (for β5i) or 100 μM (for *Mtb* proteasome) of each compound (standard errors for RAs were <15%). [b] Published previously in: Sosič, I.; Gobec, M.; Brus, B.; Knez, D.; Živec, M.; Konc, J.; Lešnik, S.; Ogrizek, M.; Obreza, A.; Žigon, D.; Janežič, D.; Mlinarič-Raščan, I.; Gobec, S., Nonpeptidic selective inhibitors of the chymotrypsin-like (β5i) subunit of the immunoproteasome. *Angew Chem Int Ed Engl* **2016,** *55* (19), 5745-8; Shannon Schiffrer, E.; Sosič, I.; Šterman, A.; Mravljak, J.; Mlinarič-Raščan, I.; Gobec, S.; Gobec, M., A focused structure–activity relationship study of psoralen-based immunoproteasome inhibitors. *Med Chem Commun* **2019**, *10* (11), 1958-65. ND, not determined.

3. Determination of K_i and K_m values

K_m for Suc-LLVY-AMC of 60 ± 15 μM was determined (See *Material and Methods* for details).

Michaelis-Menten kinetics – plot of reaction rate (v) v *vs* substrate concentration [S]

Dixon plot – plot of the reciprocal of the reaction rate (1/v) *vs* substrate concentrations [S]

Lineweaver – Burk plot – plot of the reciprocal of the reaction rate (1/v) *vs* the reciprocal of the substrate concentration [S]

a. Bortezomib

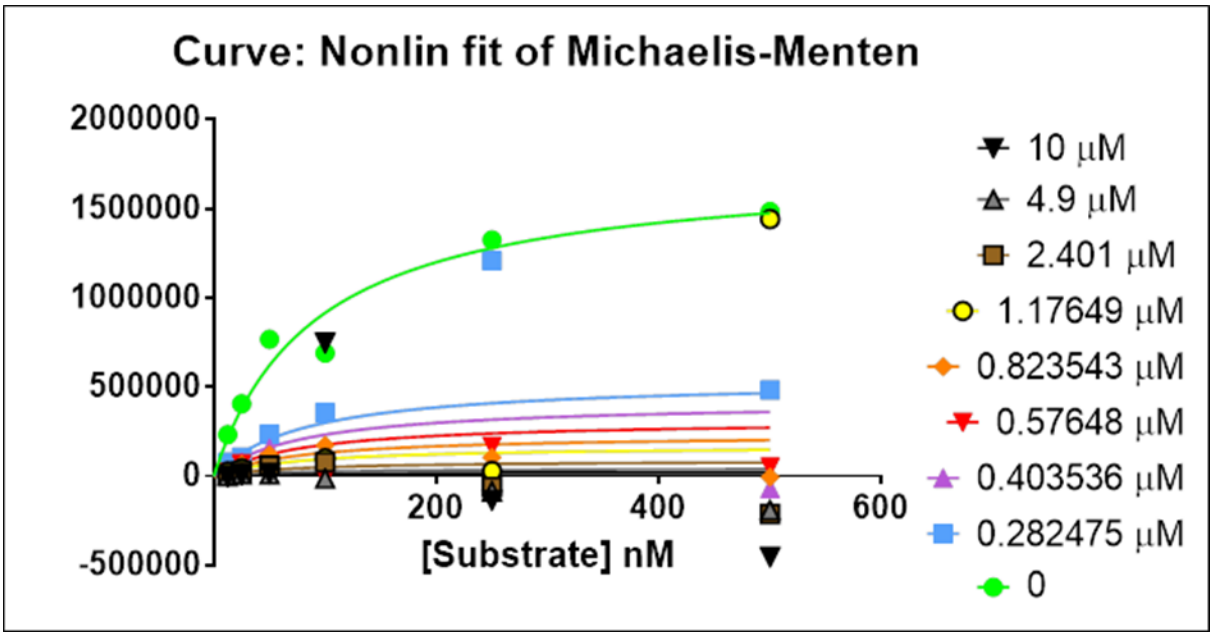

Figure S2. Michaelis-Menten plot for bortezomib based on enzyme activity (10 nM) at various concentrations of the compound (written on the right) and various concentrations of the substrate Suc-LLVY-AMC (12.5, 25, 50, 100, 250 and 500 μM).

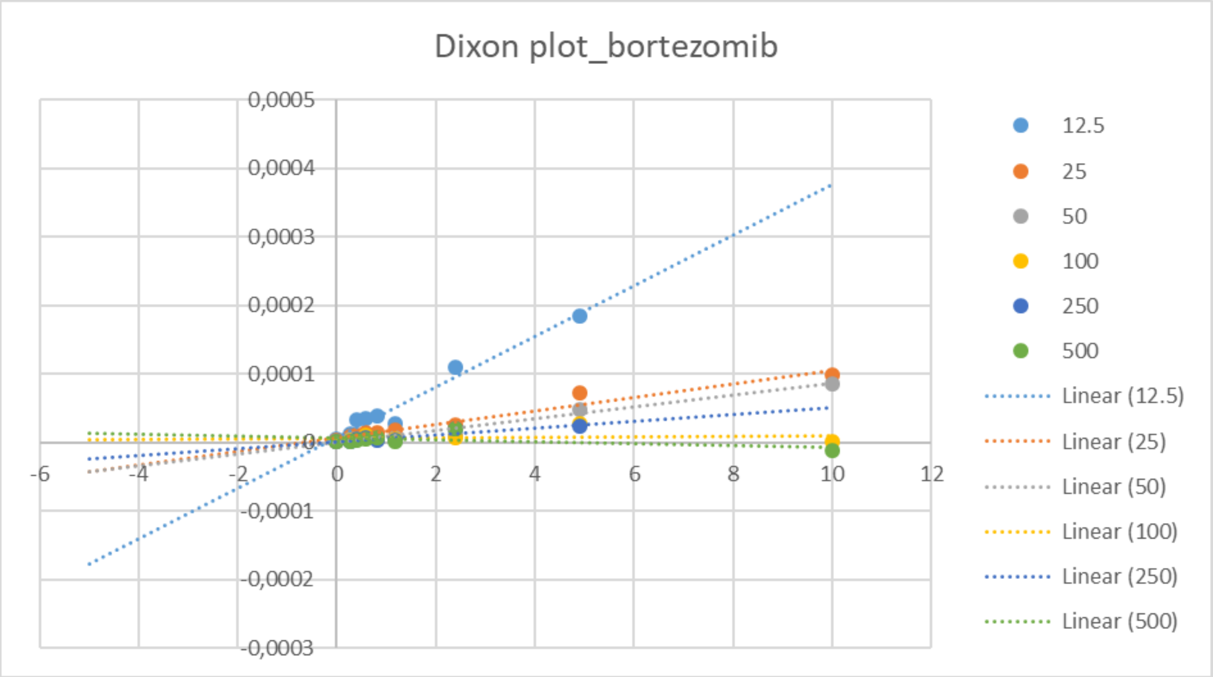

Figure S3. Dixon plot for bortezomib based on enzyme activity (10 nM) at various concentration of the substrate Suc-LLVY-AMC (12.5, 25, 50, 100, 250 and 500 μM).

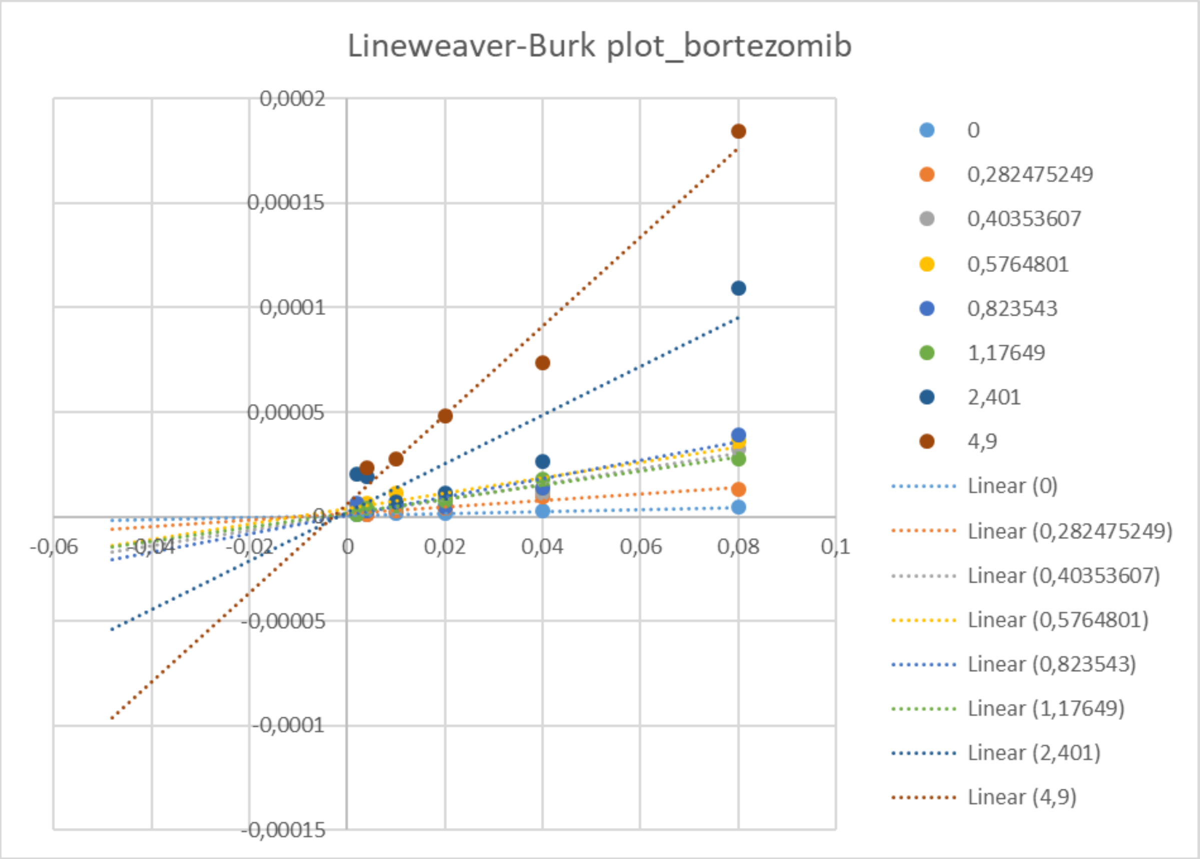

Figure S4. Lineweaver-Burk plot for bortezomib based on enzyme activity (10 nM) at various concentrations of the compound (written on the right).

b. PR-957

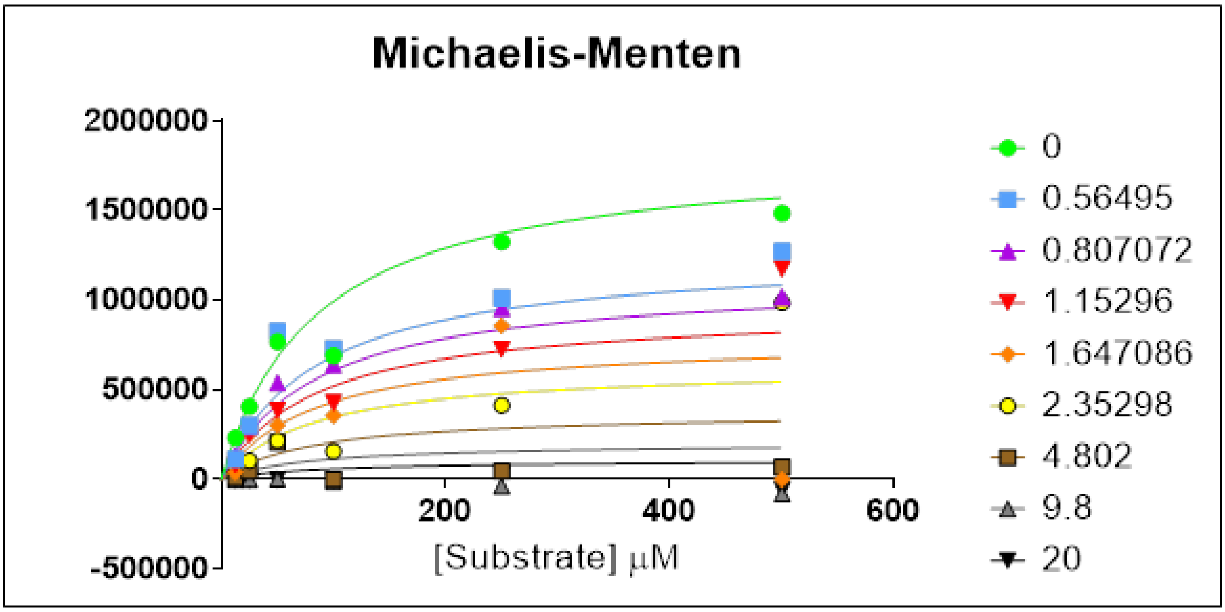

Figure S5. Michaelis-Menten plot for compound **PR-957** based on enzyme activity (10 nM) at various concentrations of the compound (written on the right) and various concentrations of the substrate Suc-LLVY-AMC (12.5, 25, 50, 100, 250 and 500 µM).

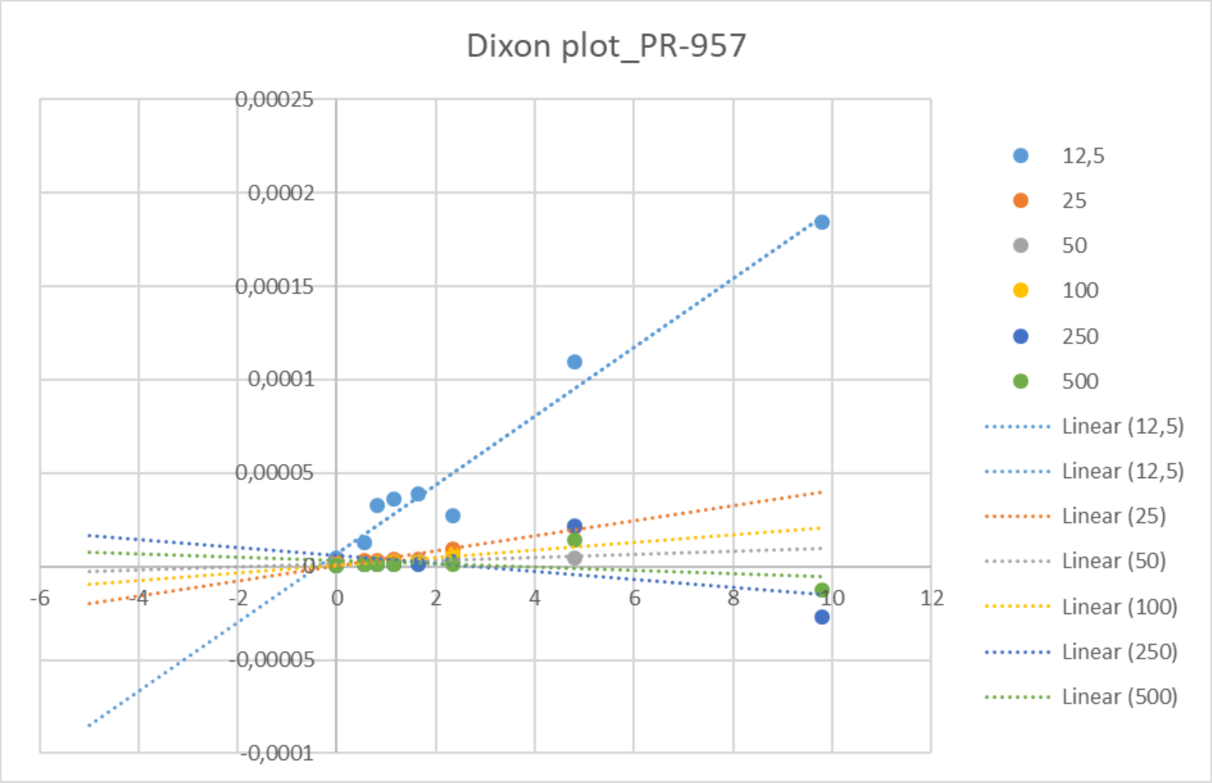

Figure S6. Dixon plot for compound **PR-957** based on enzyme activity (10 nM) at various concentration of the substrate Suc-LLVY-AMC (12.5, 25, 50, 100, 250 and 500 µM).

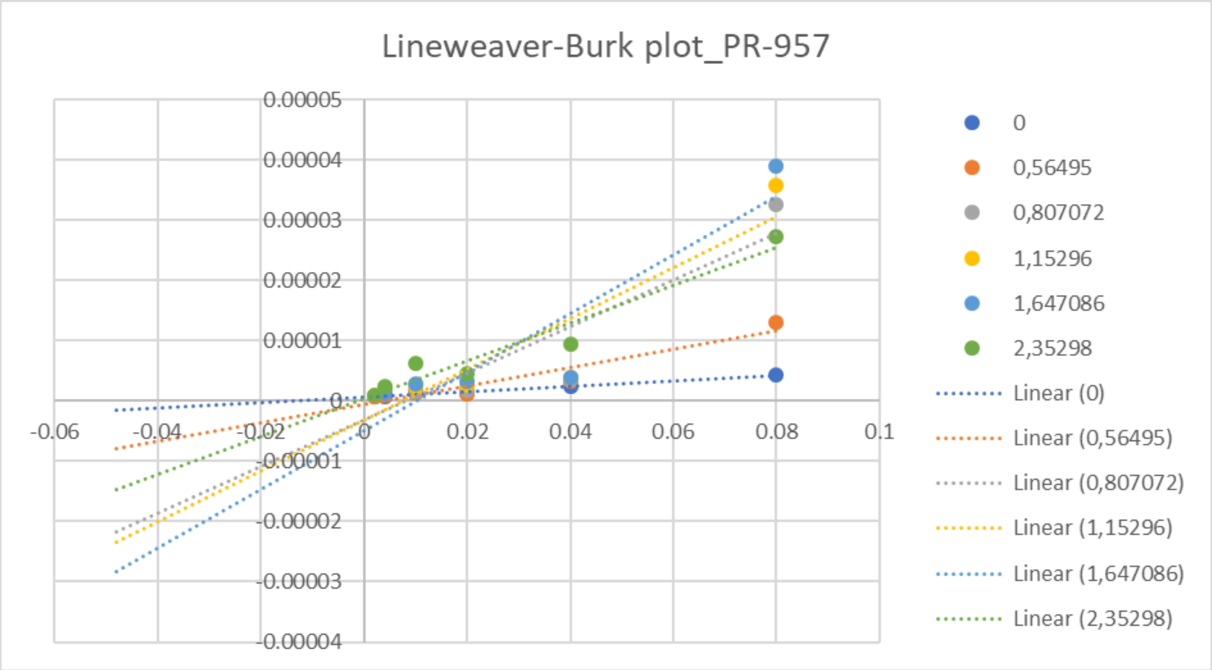

Figure S7. Lineweaver-Burk plot for compound **PR-957** based on enzyme activity (10 nM) at various concentrations of the compound (written on the right).

c. Compound 8

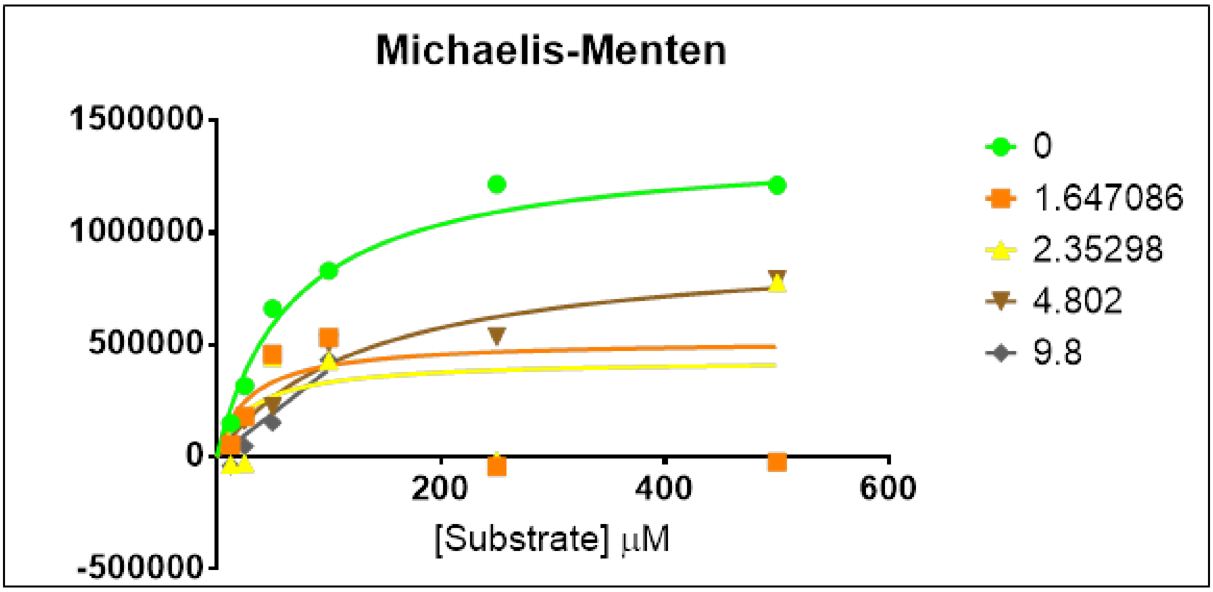

Figure S8. Michaelis-Menten plot for compound **8** based on enzyme activity (10 nM) at various concentrations of the compound (written on the right) and various concentrations of the substrate Suc-LLVY-AMC (12.5, 25, 50, 100, 250 and 500 µM).

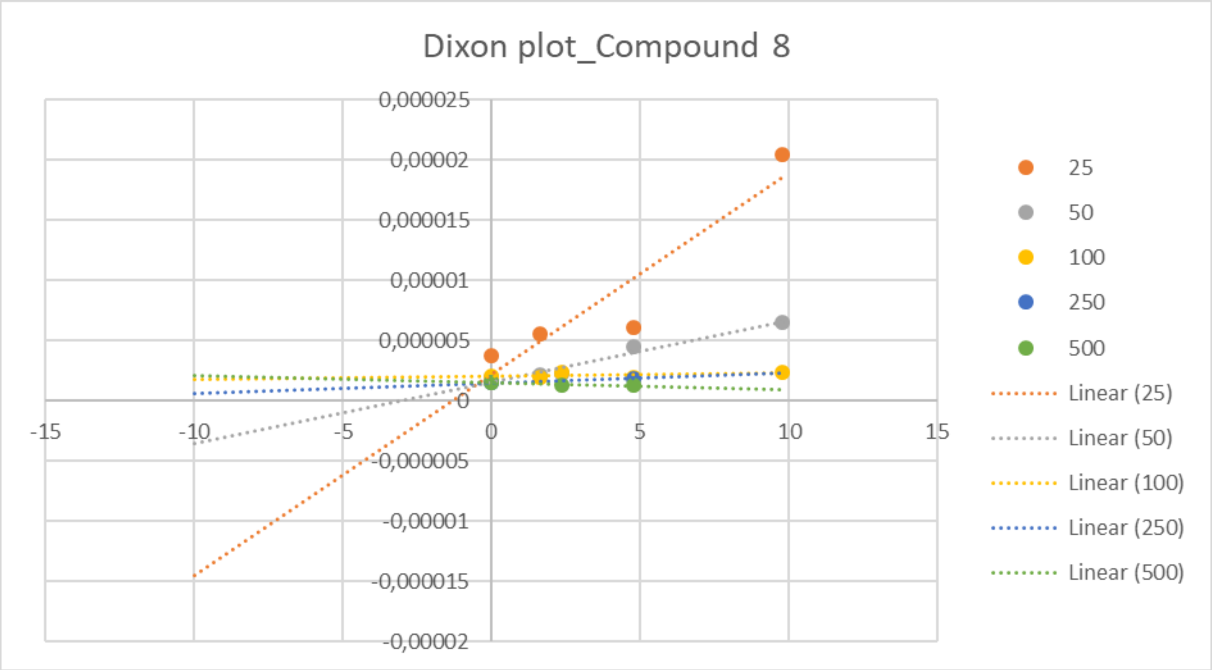

Figure S9. Dixon plot for compound **8** based on enzyme activity (10 nM) at various concentration of the substrate Suc-LLVY-AMC (12.5, 25, 50, 100, 250 and 500 µM).

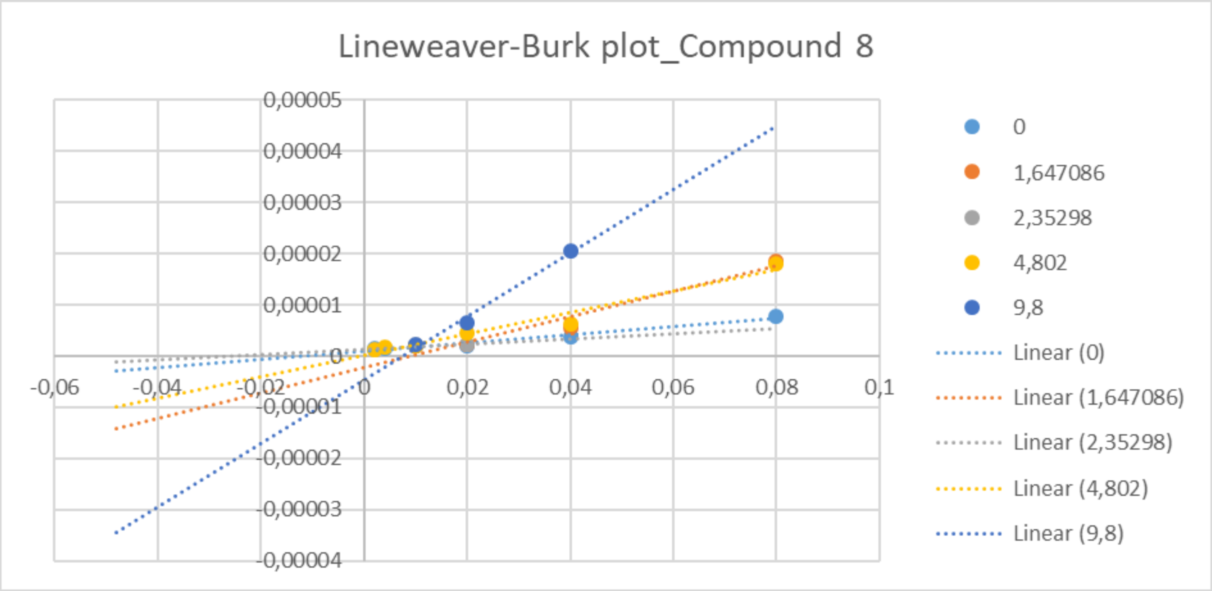

Figure S10. Lineweaver-Burk plot for compound **8** based on enzyme activity (10 nM) at various concentrations of the compound (written on the right).

d. Compound 11

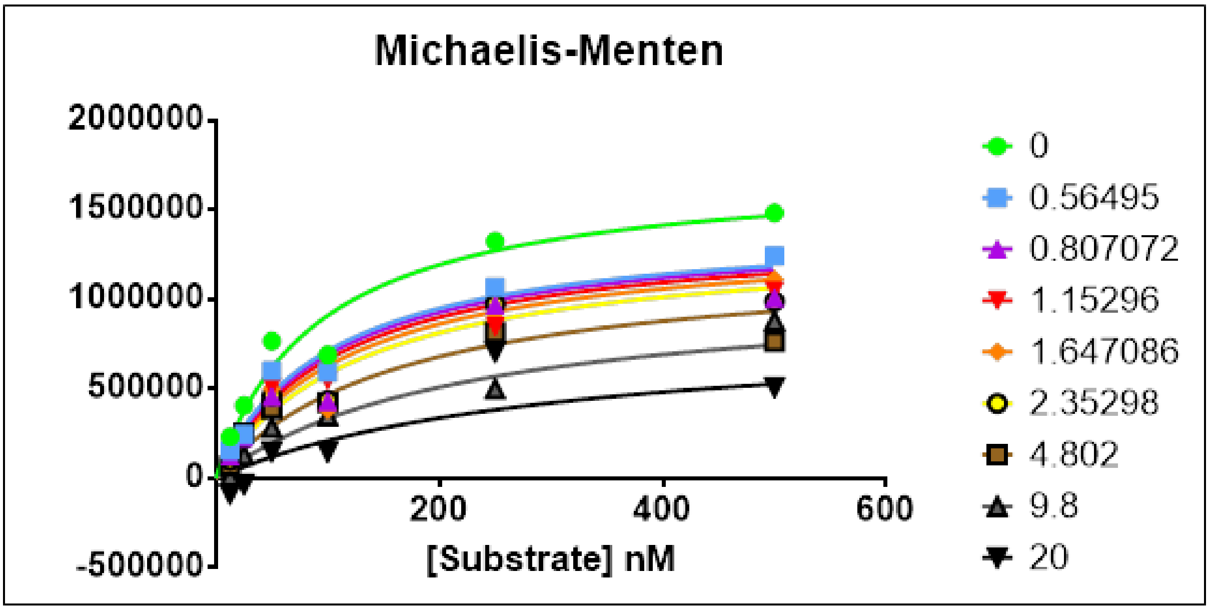

Figure S11. Michaelis-Menten plot for compound **11** based on enzyme activity (10 nM) at various concentrations of the compound (written on the right) and various concentrations of the substrate Suc-LLVY- AMC (12.5, 25, 50, 100, 250 and 500 μM).

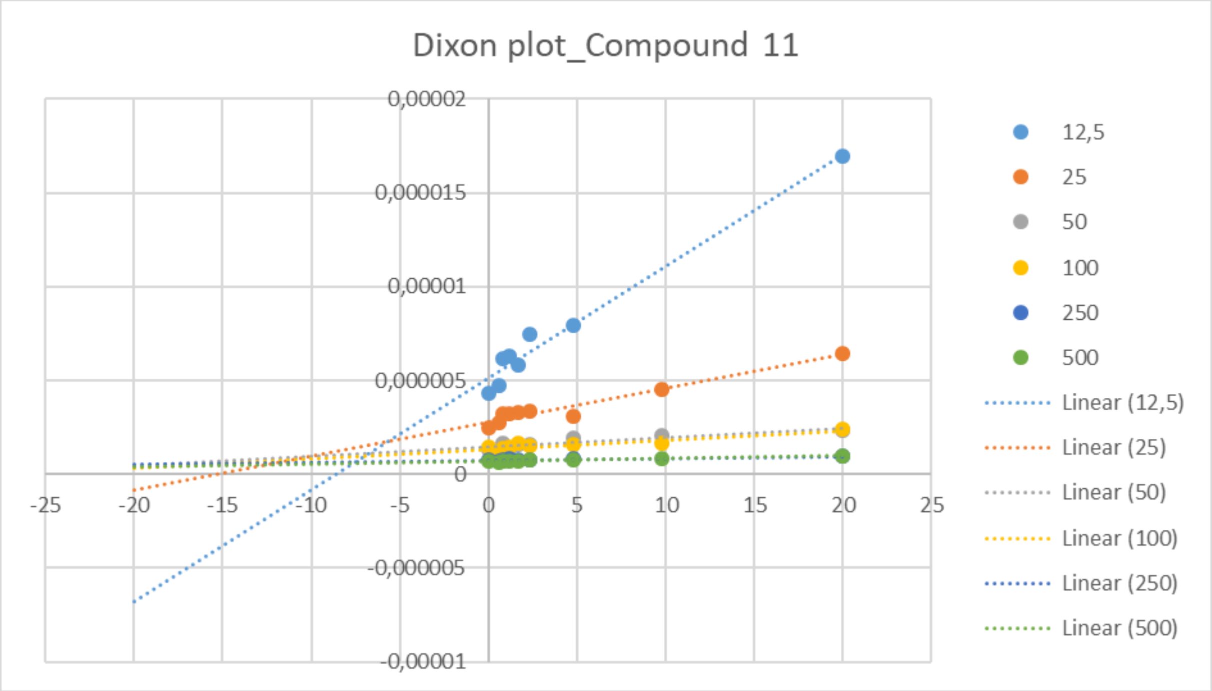

Figure S12. Dixon plot for compound **11** at various concentration of the substrate Suc-LLVY-AMC (12.5, 25, 50, 100, 250 and 500 μM).

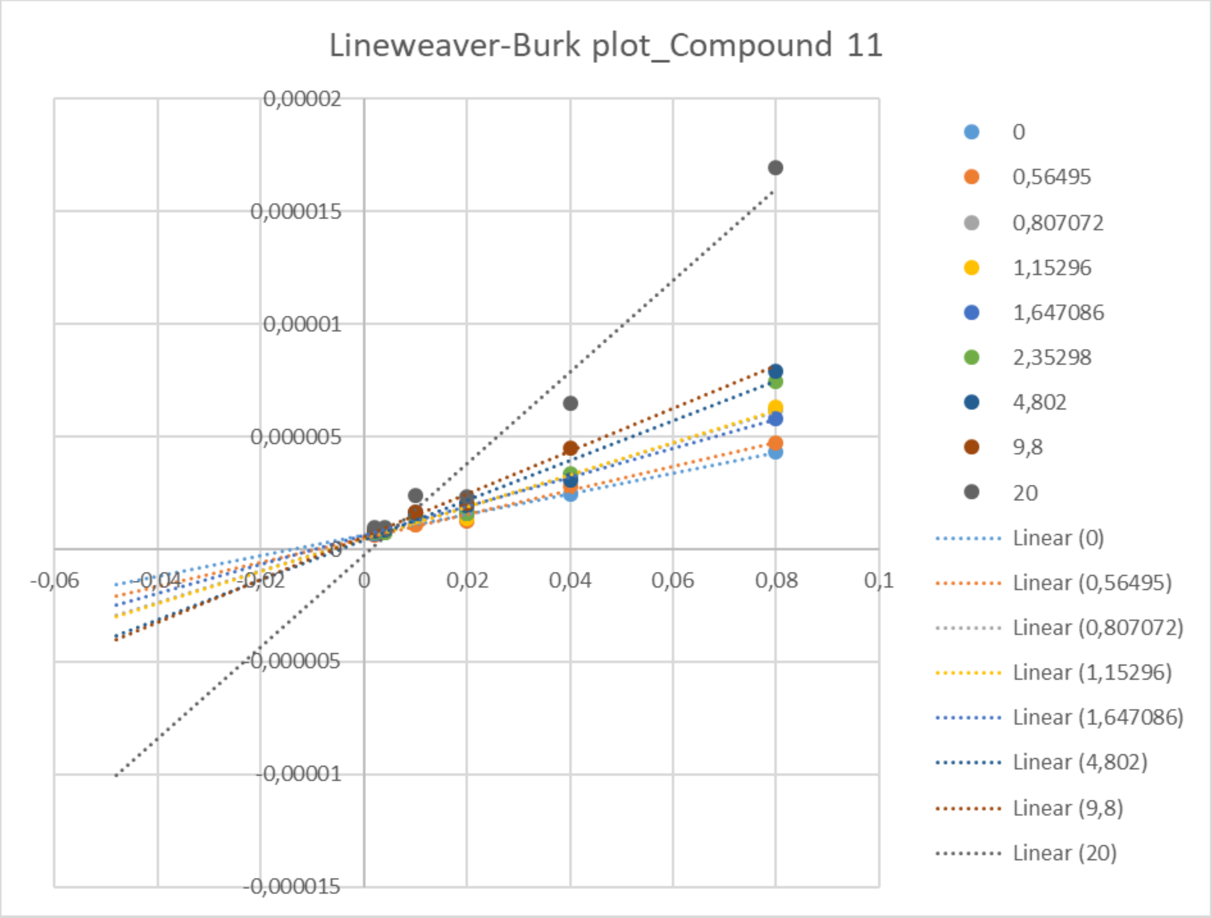

Figure S13. Lineweaver-Burk plot for compound **11** at various concentrations of the compound (written on the right).

e. Compound 13

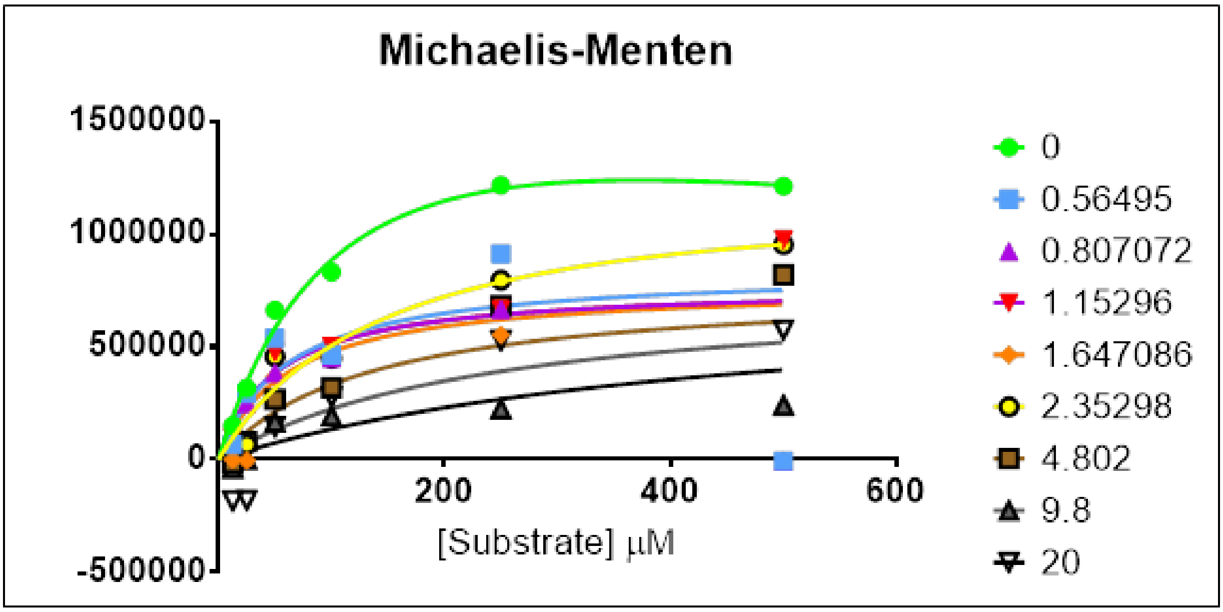

Figure S14. Michaelis-Menten plot for compound **13** based on enzyme activity (10 nM) at various concentrations of the compound (written on the right) and various concentrations of the substrate Suc-LLVY-AMC AMC (12.5, 25, 50, 100, 250 and 500 µM).

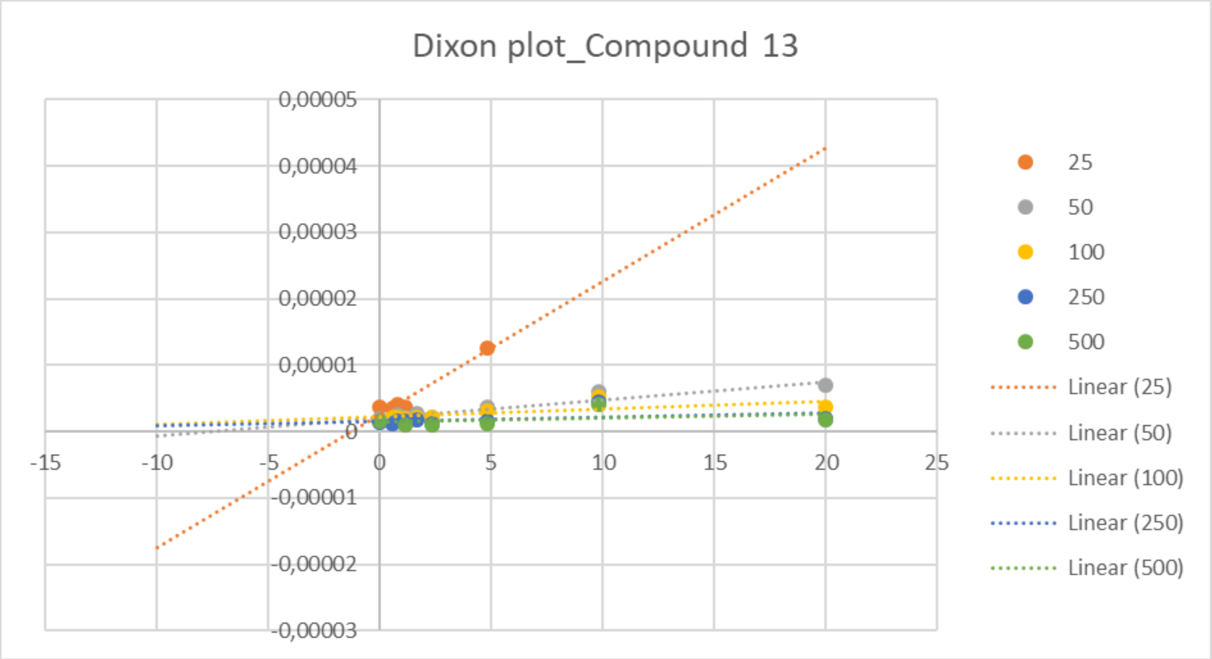

Figure S15. Dixon plot for compound **13** at various concentration of the substrate Suc-LLVY-AMC (12.5, 25, 50, 100, 250 and 500 μM).

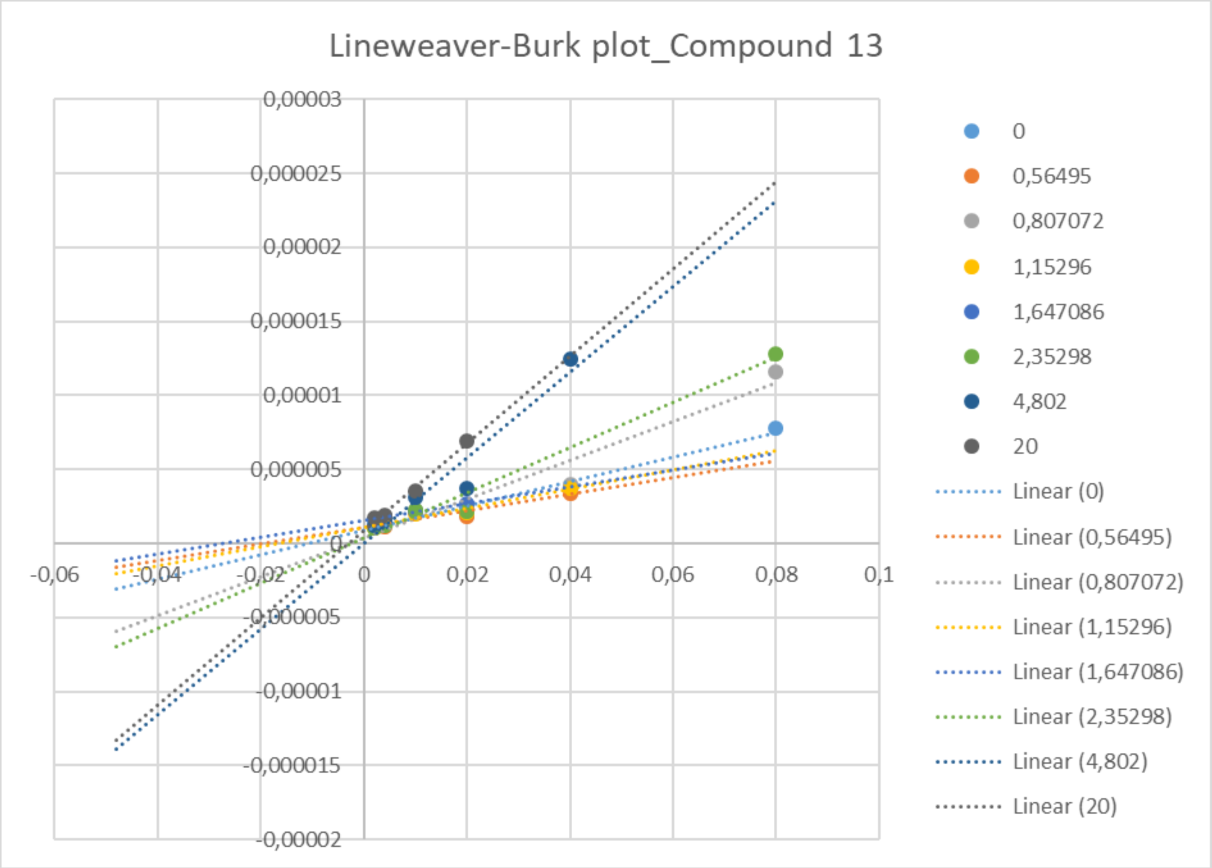

Figure S16. Lineweaver-Burk plot for compound **13** at various concentrations of the compound (written on the right).

f. Compound 15

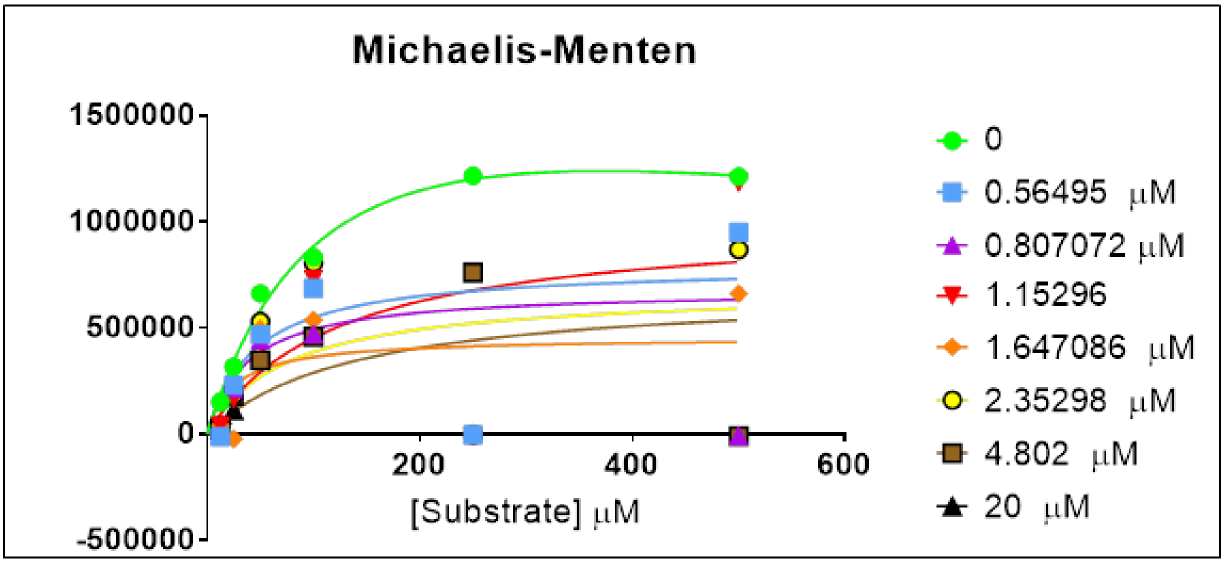

Figure S17. Michaelis-Menten plot for compound **15** based on enzyme activity (10 nM) at various concentrations of the compound (written on the right) and various concentrations of the substrate Suc-LLVY- AMC (12.5, 25, 50, 100, 250 and 500 µM).

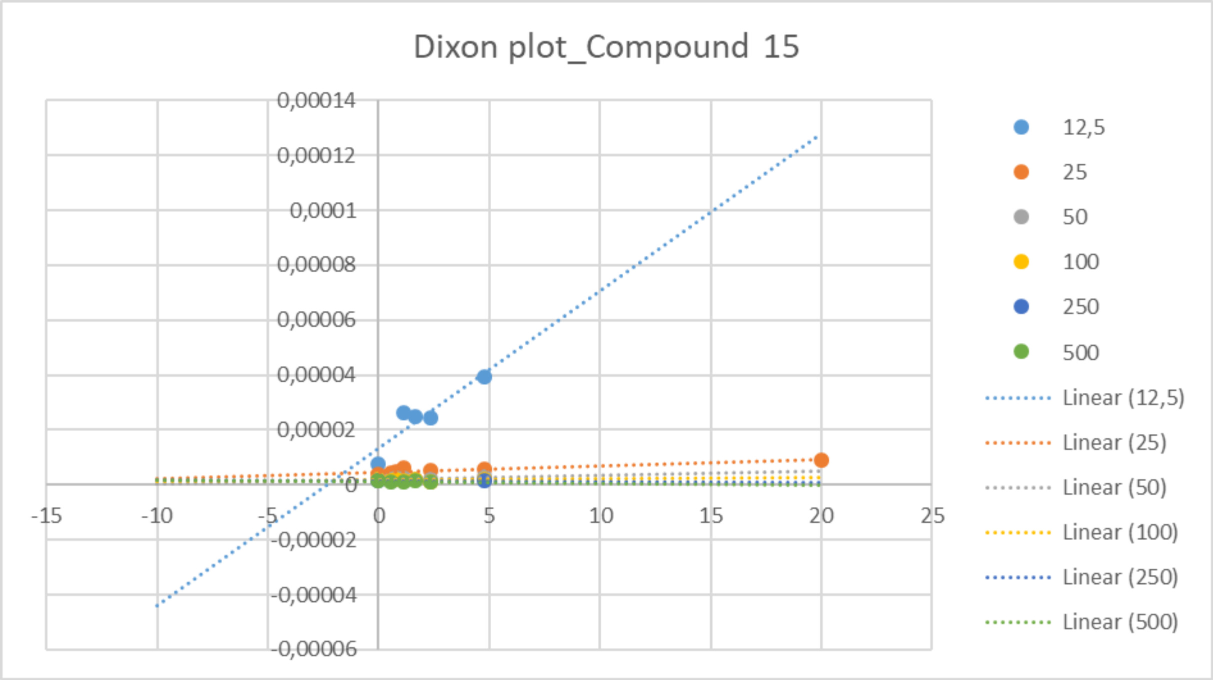

Figure S18. Dixon plot for compound **15** at various concentration of the substrate Suc-LLVY-AMC (12.5, 25, 50, 100, 250 and 500 µM).

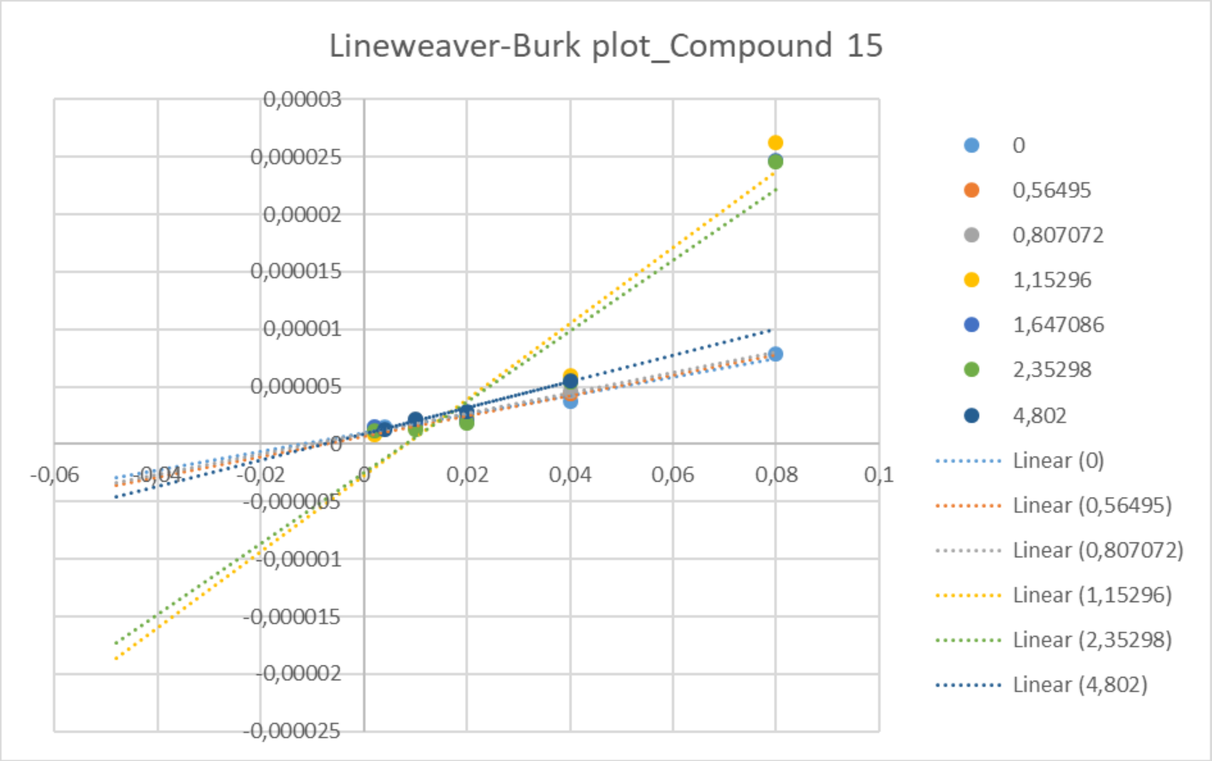

Figure S19. Lineweaver-Burk plot for compound **15** at various concentrations of the compound (written on the right).

g. Determination of K_m value for Suc-LLVY-AMC

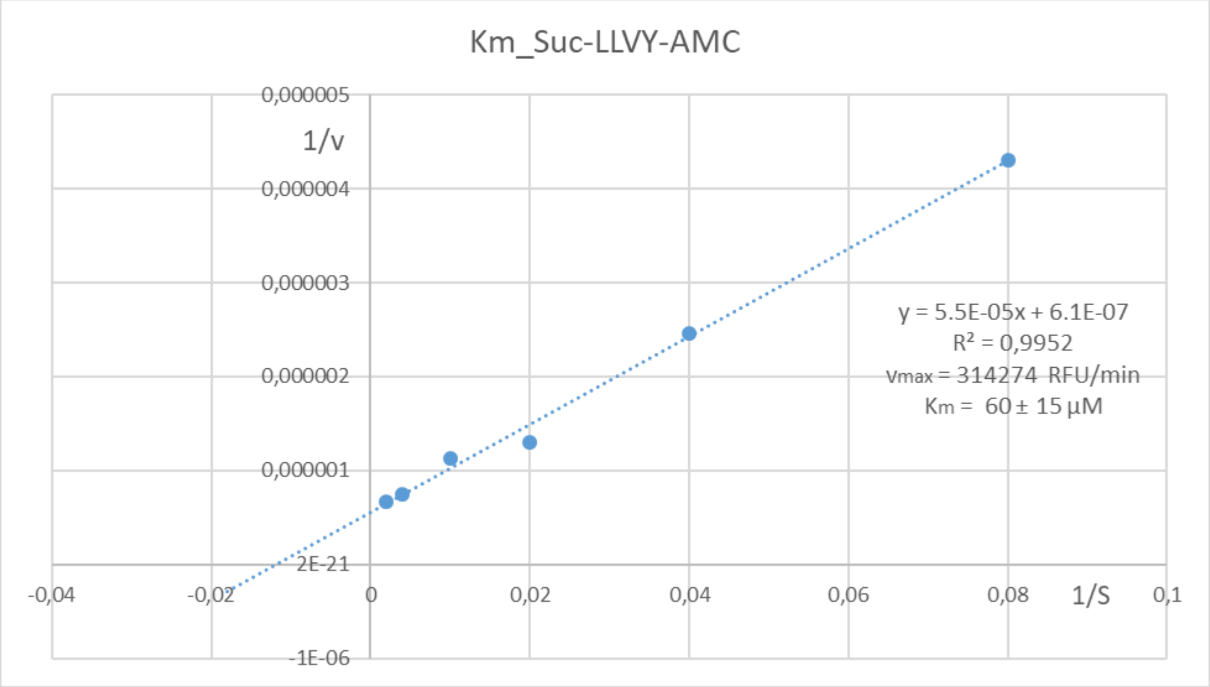

Figure S20. Lineweaver-Burk plot showing the increase in initial velocity for Mtb proteasome catalytic reaction in the presence of increasing concentration of the substrate Suc-LLVY-AMC. .

4. Dilution assay

a. Bortezomib

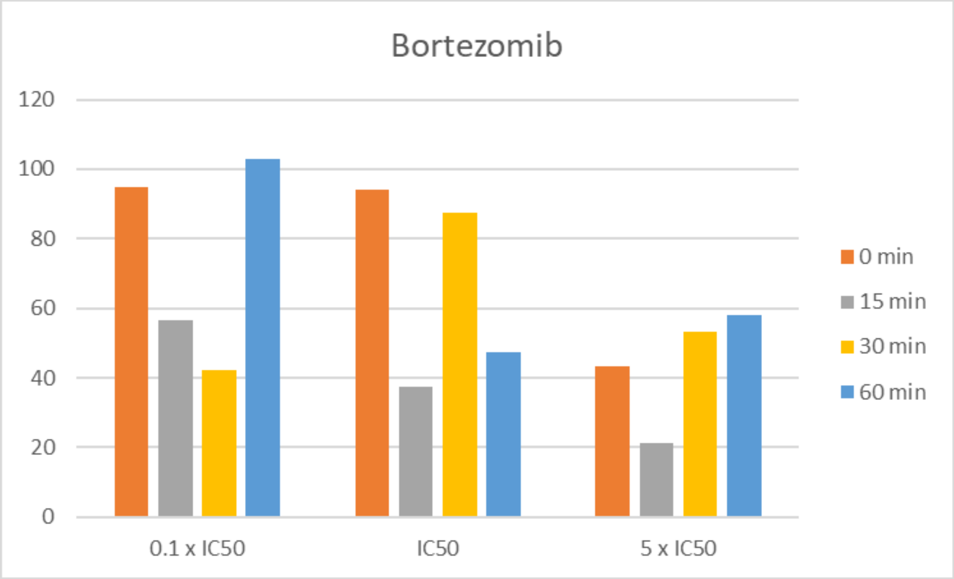

Figure S21. Restored activity (%) of Mtb proteasome after preincubation of enyzme with bortezomib at various concentrations (0.1 x IC_{50}, IC_{50} and 5 x IC_{50}) for various amounts of time (0, 15, 30 and 60 min).

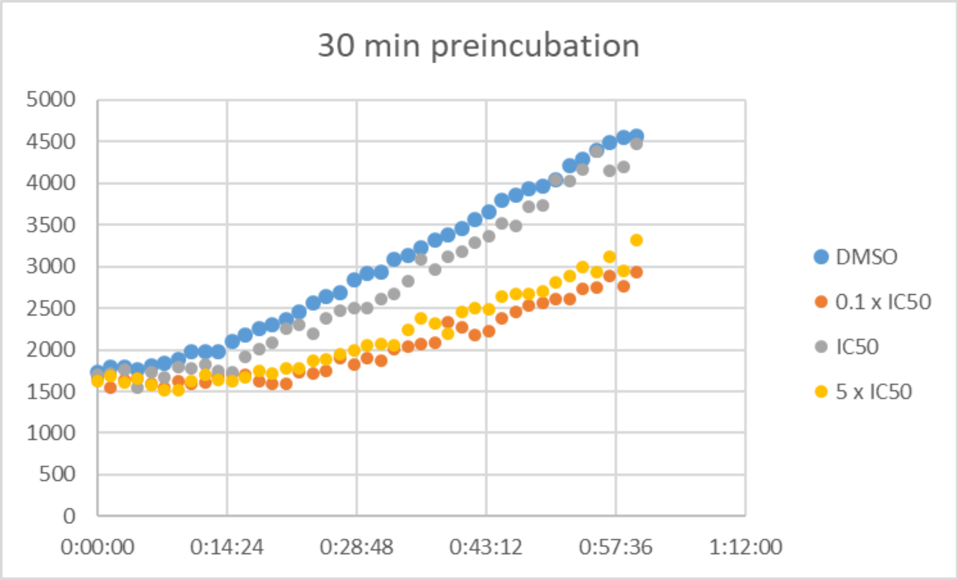

Figure S22. Restored activity (RFU) of Mtb proteasome after 30-min preincubation of enyzme with bortezomib at various concentrations (0.1 x IC_{50}, IC_{50} and 5 x IC_{50}).

b. PR-957

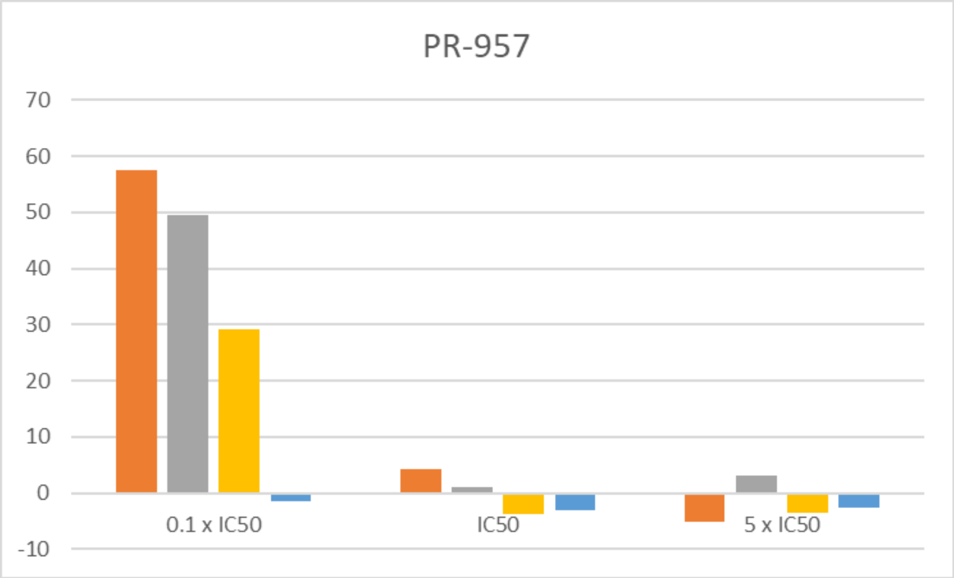

Figure S23. Restored activity (%) of Mtb proteasome after preincubation of enyzme with **PR-957** at various concentrations (0.1 x IC_{50}, IC_{50} and 5 x IC_{50}) for various amounts of time (0, 15, 30 and 60 min).

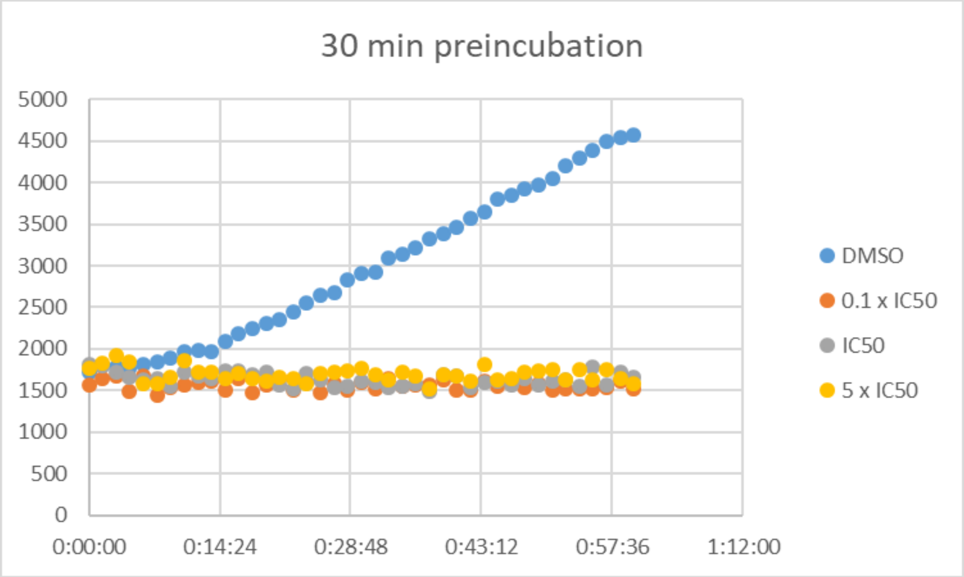

Figure S24. Restored activity (RFU) of Mtb proteasome after 30-min preincubation of enyzme with **PR-957** at various concentrations (0.1 x IC_{50}, IC_{50} and 5 x IC_{50}).

c. Compound 8

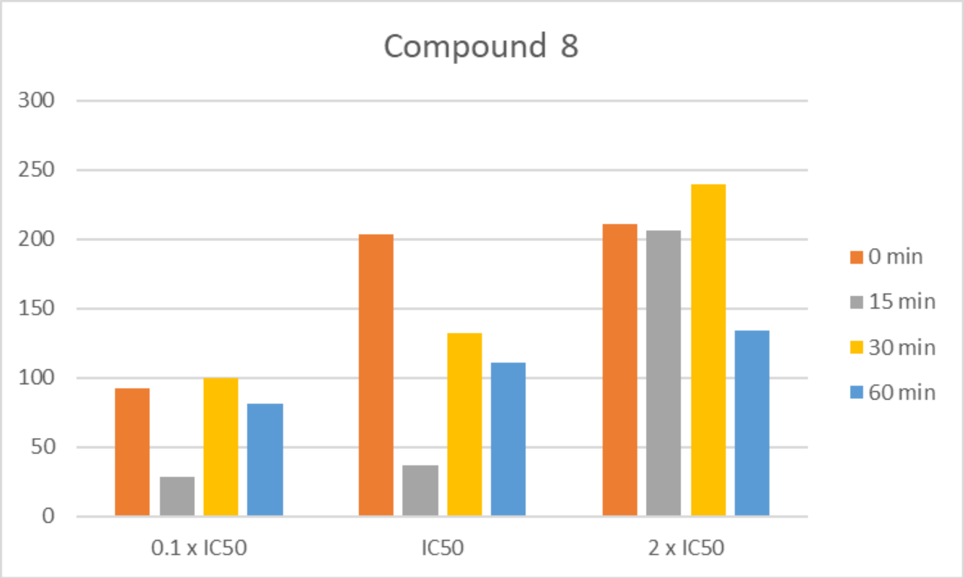

Figure S25. Restored activity (%) of Mtb proteasome after preincubation of enyzme with compound **8** at various concentrations (0.1 x IC_{50}, IC_{50} and 2 x IC_{50}) for various amounts of time (0, 15, 30 and 60 min).

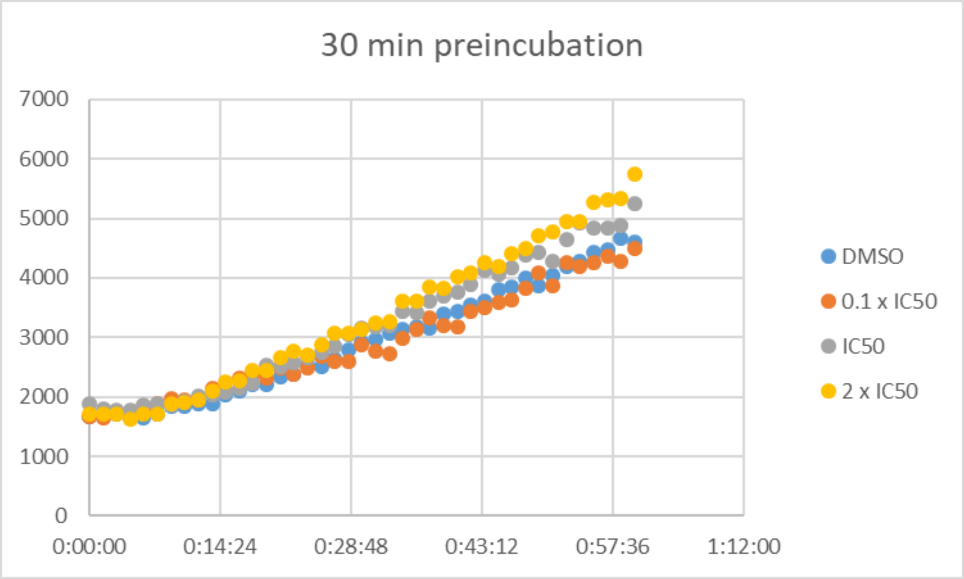

Figure S26. Restored activity (RFU) of Mtb proteasome after 30-min preincubation of enyzme with compound **8** at various concentrations (0.1 x IC_{50}, IC_{50} and 2 x IC_{50}).

d. Compound 11

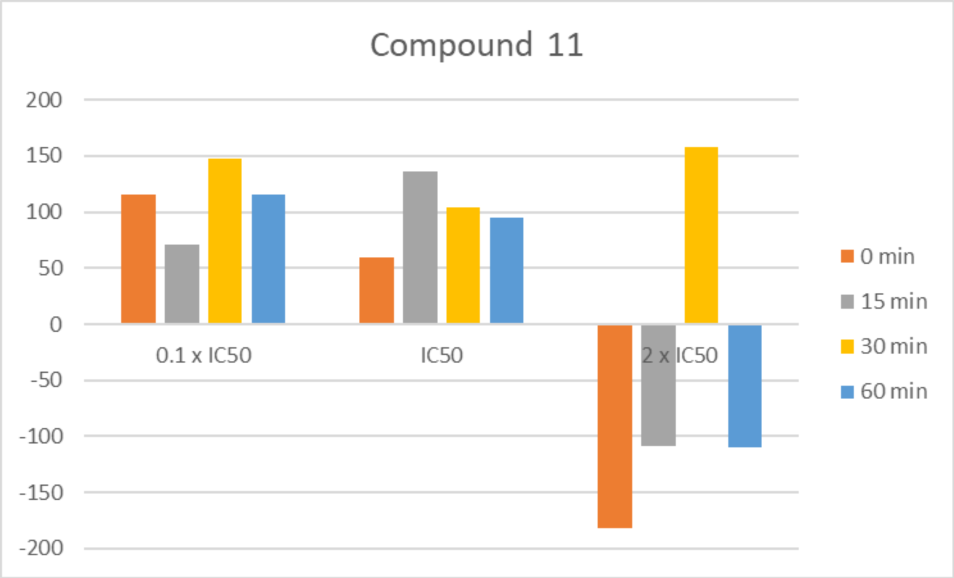

Figure S27. Restored activity (%) of Mtb proteasome after preincubation of enyzme with compound **11** at various concentrations (0.1 x IC_{50}, IC_{50} and 2 x IC_{50}) for various amounts of time (0, 15, 30 and 60 min).

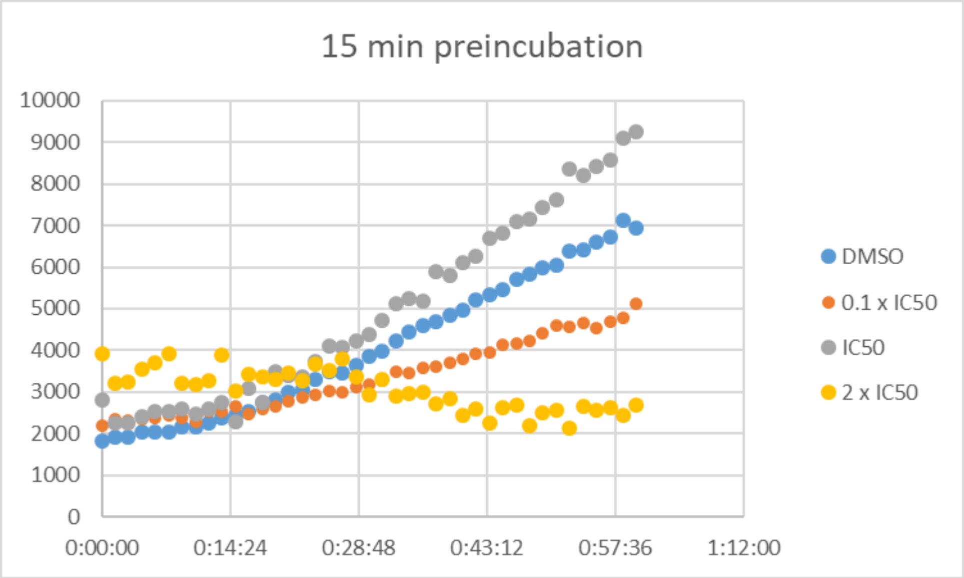

Figure S28. Restored activity (RFU) of Mtb proteasome after 15-min preincubation of enyzme with compound **11** at various concentrations (0.1 x IC_{50}, IC_{50} and 2 x IC_{50}).

e. Compound 13

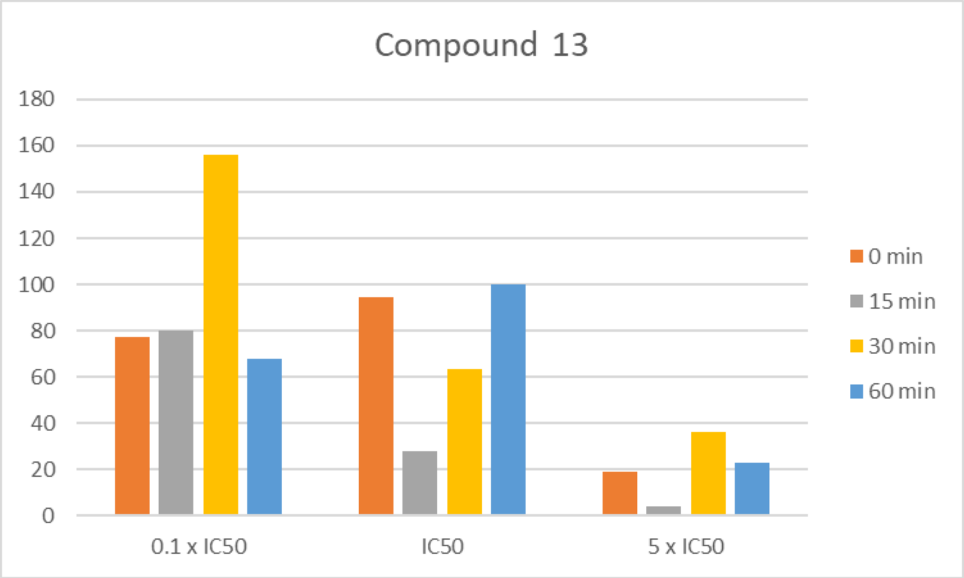

Figure S29. Restored activity (%) of Mtb proteasome after preincubation of enyzme with compound **13** at various concentrations (0.1 x IC_{50}, IC_{50} and 5 x IC_{50}) for various amounts of time (0, 15, 30 and 60 min).

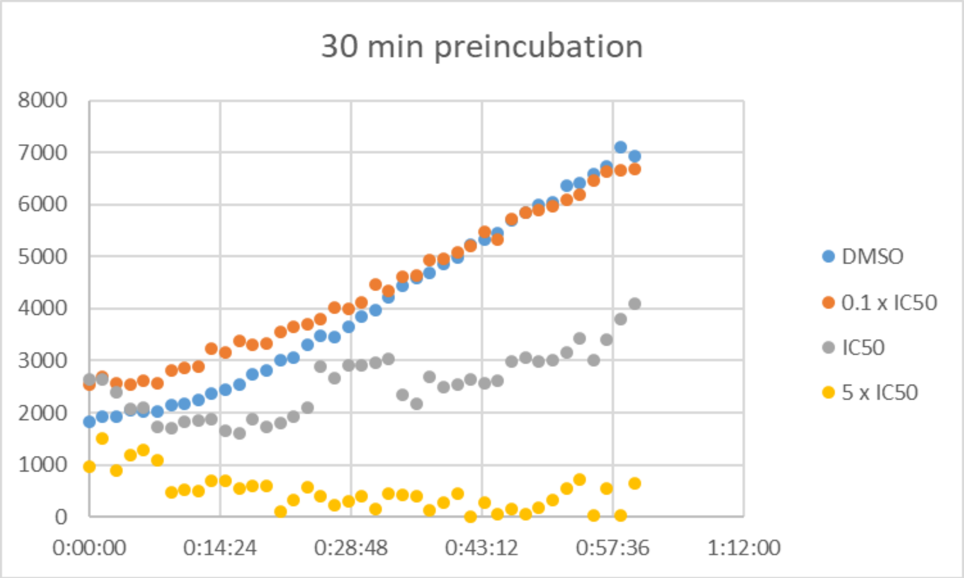

Figure S30. Restored activity (RFU) of Mtb proteasome after 30-min preincubation of enyzme with compound **13** at various concentrations (0.1 x IC_{50}, IC_{50} and 5 x IC_{50}).

f. Compound 15

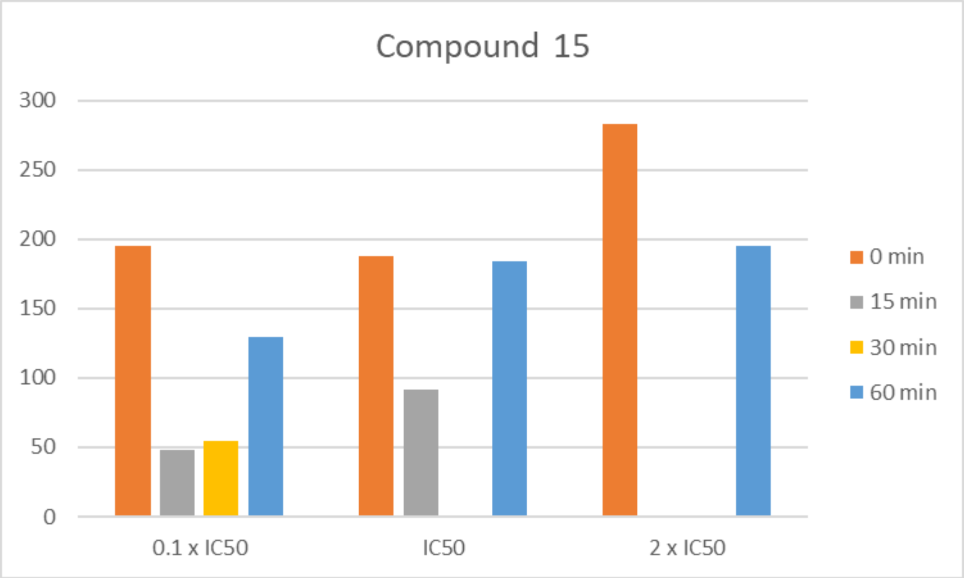

Figure S31. Restored activity (%) of Mtb proteasome after preincubation of enyzme with compound **15** at various concentrations (0.1 x IC_{50}, IC_{50} and 2 x IC_{50}) for various amounts of time (0, 15, 30 and 60 min).

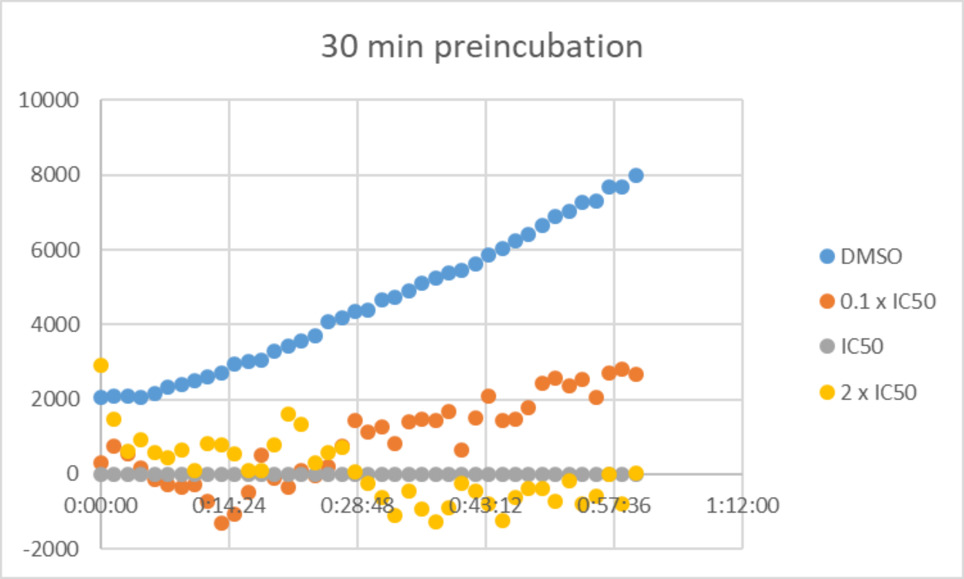

Figure S32. Restored activity (RFU) of Mtb proteasome after 30-min preincubation of enyzme with compound **15** at various concentrations (0.1 x IC_{50}, IC_{50} and 2 x IC_{50}).

5. Analytic and synthetic data for compounds

Determination of k_{inact}/K_i values for PR-957

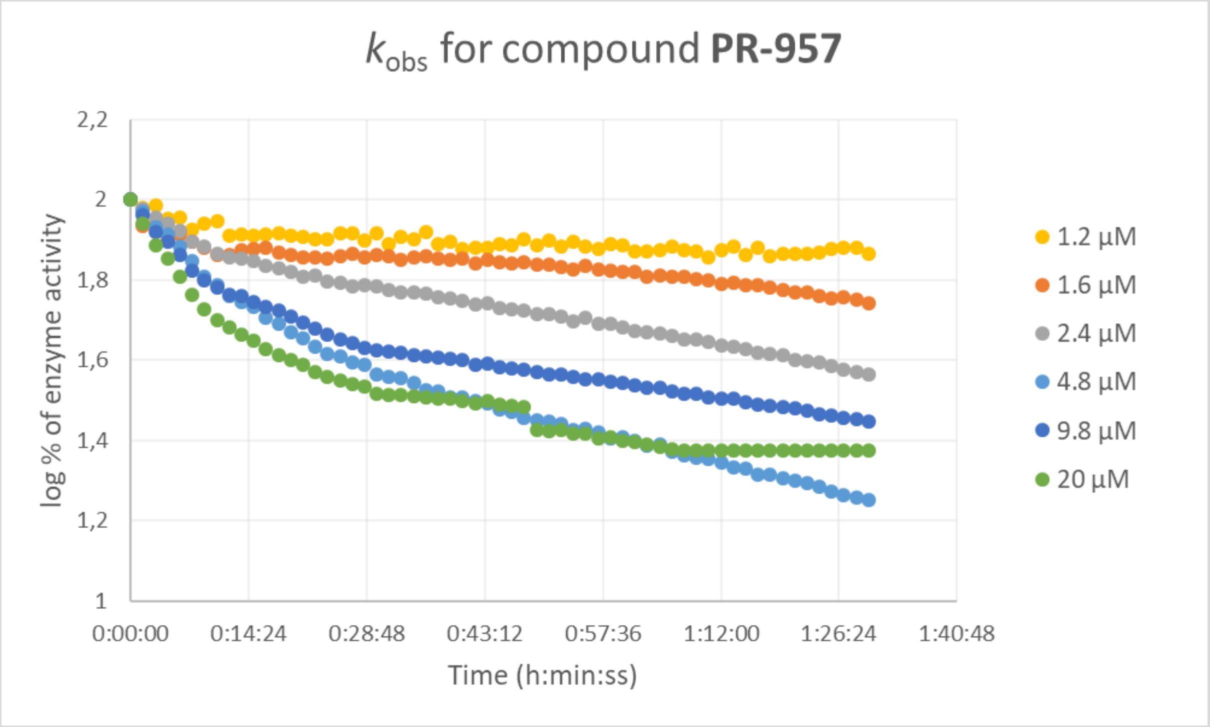

Figure S33. Plot of logaritm of % of enyzme activity vs time for compound **PR-957** during the 90-min reaction.

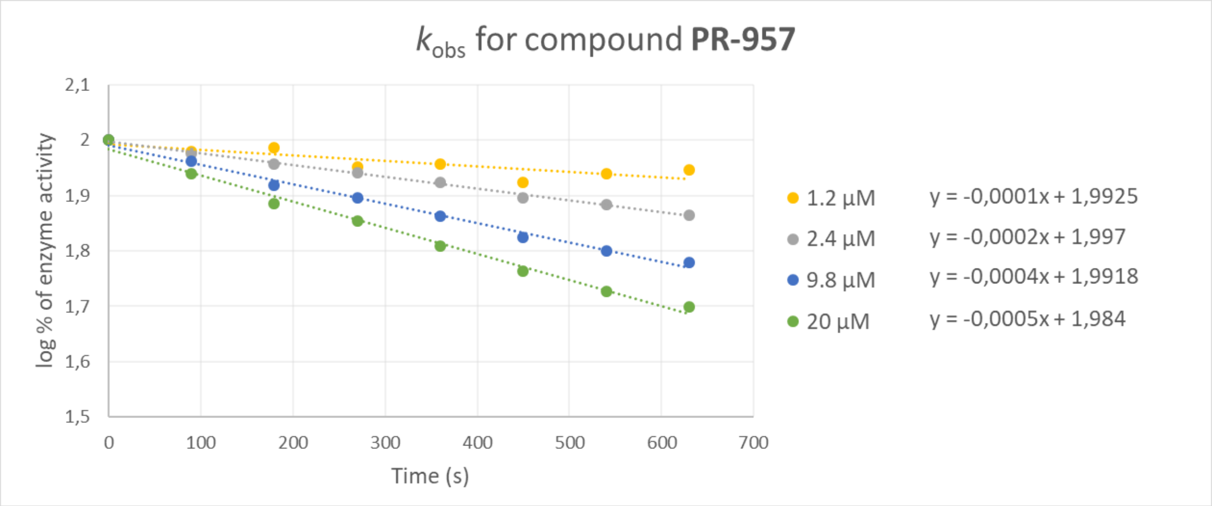

Figure S34. Determination of initial rate of the reaction, k_{obs}, by plotting logaritm of % of enzyme activity vs time (first 10 min of the reaction).

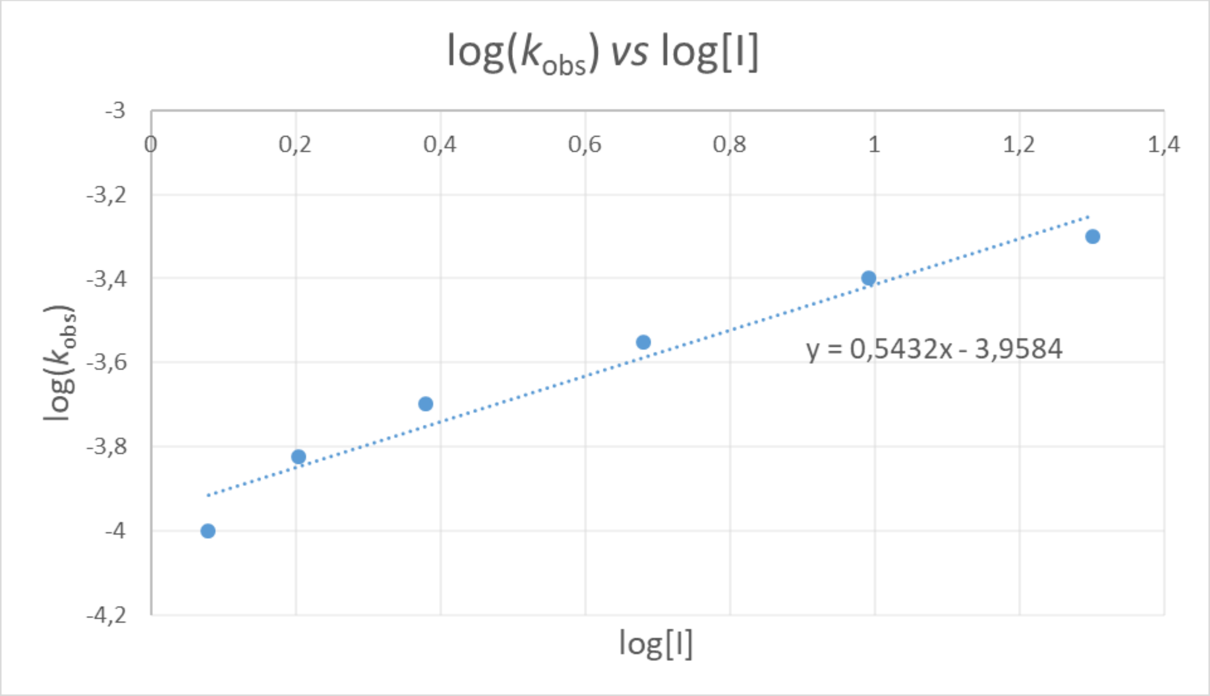

Figure S35. Plot of logaritm of k_{obs} vs logaritem of inhibitor concentration for compound **PR-957**.

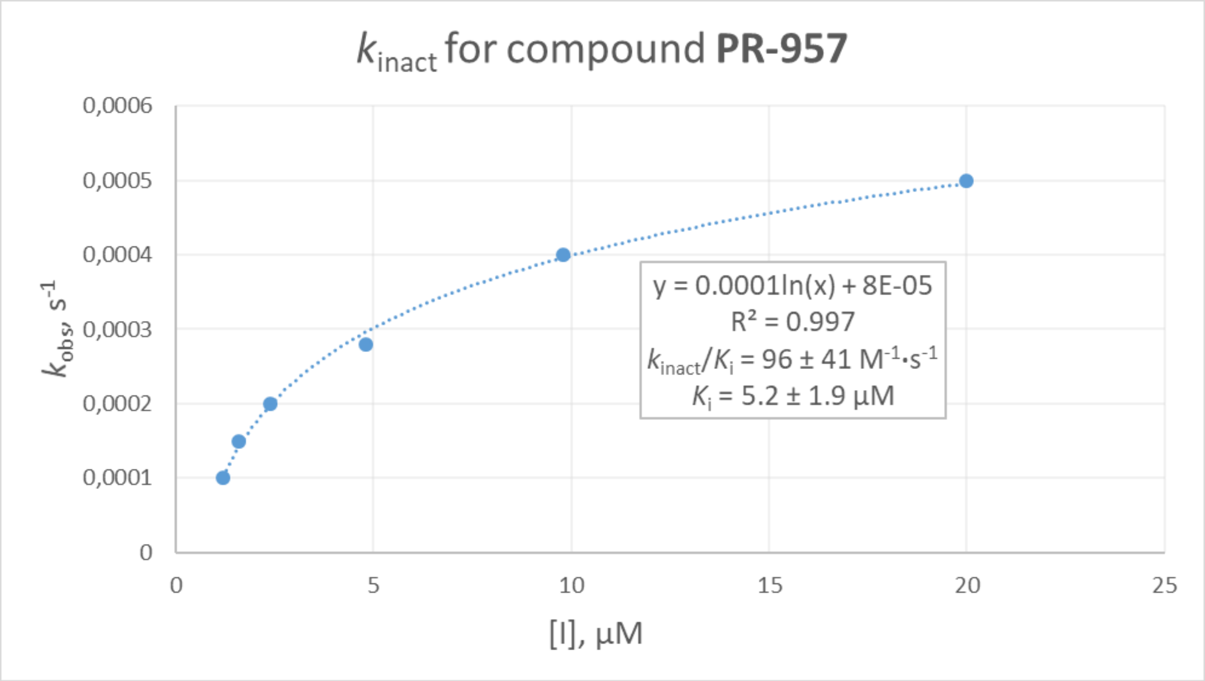

Figure S36. Determination of constant of inactivation, k_{inact}, from plot of k_{obs} vs inhibitor concentration, [I].
